# Metabolic Reprogramming Helps to Define Different Metastatic Tropisms in Colorectal Cancer

**DOI:** 10.3389/fonc.2022.903033

**Published:** 2022-07-25

**Authors:** Ana Montero-Calle, Marta Gómez de Cedrón, Adriana Quijada-Freire, Guillermo Solís-Fernández, Victoria López-Alonso, Isabel Espinosa-Salinas, Alberto Peláez-García, María Jesús Fernández-Aceñero, Ana Ramírez de Molina, Rodrigo Barderas

**Affiliations:** ^1^ Functional Proteomics Unit, Chronic Disease Programme, UFIEC, Instituto de Salud Carlos III, Madrid, Spain; ^2^ Precision Nutrition and Cancer Program, Molecular Oncology Group, IMDEA Food Institute, Campus of International Excellence (CEI) University Autonomous of Madrid (UAM) + Consejo Superior de Investigaciones Científicas (CSIC), Madrid, Spain; ^3^ Molecular Imaging and Photonics Division, Chemistry Department, Faculty of Sciences, KU Leuven, Leuven, Belgium; ^4^ Unidad de Biología Computacional, Chronic Disease Programme, UFIEC, Instituto de Salud Carlos III, Madrid, Spain; ^5^ Platform for Clinical Trials in Nutrition and Health (GENYAL), IMDEA Food Institute, Campus of International Excellence (CEI) University Autonomous of Madrid (UAM) + Consejo Superior de Investigaciones Científicas (CSIC), Madrid, Spain; ^6^ Molecular Pathology and Therapeutic Targets Group, La Paz University Hospital (IdiPAZ), Madrid, Spain; ^7^ Servicio de Anatomía Patológica Hospital Clínico San Carlos, Departamento de Anatomía Patológica, Facultad de Medicina, Complutense University of Madrid, Madrid, Spain

**Keywords:** CRC (colorectal cancer), metabolic reprograming, tropism of metastasis, obesity, fatty acids (FA), organotropism

## Abstract

Approximately 25% of colorectal cancer (CRC) patients experience systemic metastases, with the most frequent target organs being the liver and lung. Metabolic reprogramming has been recognized as one of the hallmarks of cancer. Here, metabolic and functional differences between two CRC cells with different metastatic organotropisms (metastatic KM12SM CRC cells to the liver and KM12L4a to the lung when injected in the spleen and in the tail vein of mice) were analysed in comparison to their parental non-metastatic isogenic KM12C cells, for a subsequent investigation of identified metabolic targets in CRC patients. Meta-analysis from proteomic and transcriptomic data deposited in databases, qPCR, WB, *in vitro* cell-based assays, and *in vivo* experiments were used to survey for metabolic alterations contributing to their different organotropism and for the subsequent analysis of identified metabolic markers in CRC patients. Although no changes in cell proliferation were observed between metastatic cells, KM12SM cells were highly dependent on oxidative phosphorylation at mitochondria, whereas KM12L4a cells were characterized by being more energetically efficient with lower basal respiration levels and a better redox management. Lipid metabolism-related targets were found altered in both cell lines, including *LDLR*, *CD36*, *FABP4*, *SCD*, *AGPAT1*, and *FASN*, which were also associated with the prognosis of CRC patients. Moreover, CD36 association with lung metastatic tropism of CRC cells was validated *in vivo.* Altogether, our results suggest that *LDLR*, *CD36*, *FABP4*, *SCD*, *FASN*, *LPL*, and *APOA1* metabolic targets are associated with CRC metastatic tropism to the liver or lung. These features exemplify specific metabolic adaptations for invasive cancer cells which stem at the primary tumour.

## Introduction

Colorectal cancer (CRC) is the third most common cancer and the second leading cause of cancer-related deaths worldwide. Metastasis is the last step of cancer, being the main responsible factor for morbidity and mortality. Moreover, more than 90% of mortality associated with CRC is due to metastasis (1). In CRC, approximately 25% of patients experience systemic metastases, with liver being the most common metastatic tropism followed by the lung (2). Despite major research efforts and clinical progress, there is still a great need for predictable biomarkers for metastasis and therapeutics for advanced CRC.

Cancer energy relies on metabolic editing to drive malignant transformation (3). Great effort has been made to characterize tumour metabolic phenotypes, and new oncometabolites are constantly being described. To support the high energy and macromolecule demands for rapid proliferation of cancer cells (4), in addition to the Warburg effect and known alterations in carbohydrate metabolism, it is recognized that tumours use a wide range of metabolic adaptations to sustain their growth and dissemination (5–7).

Metabolic changes in cancer cells are often related to growth and survival pathways that drive different aspects of tumorigenesis. For instance, glycolytic behaviour is associated with Akt and Erk pathways (8, 9), while the Myc oncogene governs glutamine addiction (10). Catabolic and anabolic alterations in lipid metabolism are also part of the metabolic reprogramming that occurs in tumour cells in response to gene mutations, loss of tumour suppressors, and/or epigenetic modifications (11). The cross talk between metabolic and tumorigenic pathways can lead to the activation of new metabolic cascades that may affect cell cycle regulation, redox management, and other changes, which in turn would enable different characteristics of tumoural cells (12). For example, ROS production, aerobic glycolysis metabolites, and the accumulation of other by-products from cancer metabolism have been shown to enhance the dissemination of cancer cells through the activation of the epithelial to mesenchymal transition (EMT) program (13–15).

In the context of cancer dissemination, a shift to a more mesenchymal state within the EMT program is thought to contribute to the early stages of metastatic translocation, i.e., tumour invasion, extravasation, release of circulating tumour cells (CTCs), and survival in the bloodstream and formation of metastatic niches. In contrast, the reverse process (mesenchymal to epithelial transition, MET) has been associated with an increased ability to proliferate and metastasize to secondary organs, which contribute to the fatal late stages of metastatic development (16). Lastly, the existence of a phenotypic plasticity is suggested to, together with the characteristics of stem cells, favour not only dissemination but also subsequent metastatic colonization (17).

Multiple studies support a good correlation between the findings observed in the KM12 cell system of CRC metastasis and patient samples, indicating that isogenic KM12 cell lines effectively recapitulate the critical steps of CRC metastasis (18–20). The in-depth proteomic characterization of the KM12 cell system, composed of the low metastatic KM12C cells and the metastatic to liver KM12SM cells, showed differences in multiple proteins, complexes, and pathways (21–23), including many metabolic pathways (22, 23). Furthermore, it was also suggested, based on the protein profile comparison of KM12SM and KM12C cells, that a partial EMT reversion could be observed in the liver metastatic CRC cells contributing to the liver colonization of these cells (22–24). Here, we aim to dive into the metabolic profile, cell bioenergetics, and metabolic genes of the two isogenic KM12 cell lines with organ-specific tropisms compared to their non-metastatic isogenic parental cell line KM12C, together with the analysis of specific alterations in EMT markers that could also contribute to the different tropisms. After being injected in spleen, KM12SM cells produce liver metastasis and KM12L4a cells show lung and liver metastasis. Furthermore, when metastatic KM12 cells are injected through the tail vein, only KM12L4a cells can metastasize to the lung.

Herein, we describe differences in the metabolic pathways and bioenergetic profiles of KM12SM and KM12L4a cells that appear to help define intrinsic protumorigenic features associated with differences in their organ-specific tropism. We found that metastatic dysregulation of *LDLR*, *CD36*, *FABP4*, *SCD*, *FASN*, *LPL*, and *APOA1* shows concordance with CRC cell tropism toward the liver versus lung, whereas *LDLR*, *CD36*, *FABP4*, *SCD*, *AGPAT1*, and *FASN* alterations are associated with CRC patients’ prognosis. In conclusion, we describe metabolic differences, which not only could help dictate the different tropism of cancer cells but also are associated with prognosis of CRC patients.

## Materials and Methods

### Proteomic Data Analysis

SILAC experiment datasets of the subcellular fractions and secretome analysis of KM12C and KM12SM cells were obtained from previous reports (22, 23). Data analysis of proteins related to metabolism and MET was performed using Gene Ontology ( http://geneontology.org), STRING (https://string-db.org), and DAVID (https://david.ncifcrf.gov) databases (**Table S1**).

### Cell Culture and Reagents

Cell lines, obtained from ATCC (ATCC, Manassas, VA, USA), were cultured in Dulbecco’s Modified Eagle’s Medium (DMEM, Lonza) supplemented with 10% foetal bovine serum (FBS, Saint Louis, Missouri, USA), 1× L-glutamine (Lonza, Basel, Switzerland), and 1× penicillin/streptavidin (Lonza), and maintained under standard conditions. N-Acetyl cysteine (NAC) and metformin were purchased form Sigma-Aldrich (Sigma-Aldrich, St. Louis, MO, USA). Images were captured using a Leica DM IL microscope (Leica Microsystems, Wetzlar, Germany), with a 10× Plan Fluotar objective and registered using Leica Application Suite (LAS).

### Analysis of Superoxide Anion and Membrane Potential

SO^*^ levels were determined with MitoSOX Red (Invitrogen Molecular Probes, Madrid, Spain; M36008) and the membrane potential quantified after staining with a TMRN probe, as previously described (25). Briefly, 10^5^ cells were seeded in a 12-well plate and treated with the probes for 30 min. After washing with PBS, cells were harvested and stained with propidium iodide to identify dead cells.

### Quantitative Real-Time PCR

RNA (1 µg) was reverse-transcribed using the High Capacity RNA-to-cDNA Master Mix system (Life Technologies, Carlsbad CA, USA) and the corresponding forward and reverse oligonucleotides (**Table S2**). Quantitative real-time PCR (qPCR) was performed with VeriQuest SYBR Green qPCR Master Mix (Affymetrix, Santa Clara, CA, USA) on the 7900HT Real-Time PCR System (Life Technologies). Relative gene expression was calculated using the 2^−ΔΔCt^ method.

### Cell-Based Assays

For proliferation analysis, MTT reagent (Sigma-Aldrich) was used for cell proliferation assays. CRC cells were harvested with Trypsin-EDTA (Lonza), and 1 × 10^4^ cells per well were seeded in 96-well plates (Corning) in 10% FBS DMEM, for 24 h at 37°C and 5% CO_2_. Then, the culture medium was removed and 150 µl of 10% FBS DMEM was added to each well. Next, cells were treated with the corresponding drugs, including control wells without any drug, in quadruplicate. For NAC assays, final NAC concentrations of 5, 10, 15, and 20 mM were tested from a 0.5-M NAC solution in H_2_O. For metformin assays, final metformin concentrations of 5 and 10 mM from a 100-mM solution in H_2_O were tested. Cells were incubated for 72 h at 37°C and 5% CO_2_, and the culture media with the corresponding drugs were changed every 24 h. After 72 h of incubation, the growth medium was removed, and wells were washed three times with 200 µl of PBS 1× (Lonza) to remove traces of drugs that may interact with the MTT reagent. Next, 100 µl of DMEM was added and, subsequently, 50 µl of 3 mg/ml MTT solution in DMEM was added to each well to a final concentration of 1 mg/ml of MTT per well. Plates were then incubated at 37°C and 5% CO_2_ for 1 h to allow cells to take up the MTT. Next, DMEM was removed and cells were lysed with 50 µl of 100% DMSO (Darmstadt, Germany). Plates were incubated with shaking during 15 min at room temperature, and finally, the absorbance at 570 nm was read with the Spark multimode microplate (TECAN, Mannedorf, Switzerland).

The migratory potential of the cells was assessed by wound-healing assays using two-well silicone inserts (Ibidi, Gräfelfing, Germany), which form a wound (gap) in the well of 500-µm width. First, silicone inserts were placed over the wells of a 24-well plate (Corning, Tewksbury, MA, USA). Then, CRC cells were harvested with Trypsin-EDTA (Lonza), and 2 × 10^5^ cells were resuspended in 70 µl of 10% FBS DMEM, seeded into each well of the silicone inserts, and incubated overnight at 37°C and 5% CO_2_. The next day, the silicone inserts were removed and 1 ml of 10% FBS DMEM was added to each well. At this point, cells were treated with or without NAC at 5 or 10 mM from a 1-M NAC solution in H_2_O, or 5 and 10 mM of metformin from a 100-mM solution in H_2_O, in duplicate. Then, the 24-well plate was placed on the TCS SP5 Confocal microscope (Leica) at 37°C and 5% CO_2_, and the size of the wound was monitored by taking photos every 2 for 62 h of each well. Finally, the images were processed with the ImageJ program (Fiji) and the MRI Wound Healing Tool.

The adhesion capacity of CRC cells was evaluated using a Matrigel matrix (Sigma-Aldrich). First, CRC cells were incubated in DMEM without FBS for 24 h at 37°C and 5% CO_2_. At the same time, 96-well plates (Corning) were coated with 100 µl Matrigel matrix (0.4 µg/mm^2^), diluted in 0.1 M NaHCO_3_, and incubated at 4°C O/N. Then, 96-well plates were blocked with 200 µl of sterile adhesion medium (DMEM 0.5% BSA) for 2 h at 37°C and CRC cells were fluorescently stained with 10 µl of 1 mg/ml BCEBF (Sigma-Aldrich) per 1 ml of DMEM for 30 min at 37°C and 5% CO_2_. Then, cells were harvested with 4 mM EDTA–PBS, and 1 × 10^5^ cells were resuspended in 50 µl of sterile adhesion medium and transferred to each precoated well, previously removing the adhesion medium. At this point, cells were treated with or without 5 or 10 mM of NAC and 5 or 10 mM of metformin from a 0.5-M and 100-mM solution of NAC or metformin, respectively, diluted in H_2_O, in quadruplicate. Subsequently, the cells were incubated for 2 h at 37°C and 5% CO_2_, and non-adhered cells were removed by decantation. Wells were washed twice with 100 µl PBS 1× to adequately remove all non-adhered cells, and finally, adhered cells were lysed with 50 µl of 10% SDS in PBS. Plates were incubated on shake for 20 min at room temperature and in the dark. The fluorescence signal was read with the Spark multimode microplate (TECAN), at 436–535-nm excitation–emission, respectively.

The invasion potential of CRC cells was evaluated using 6.5-mm transwells with 8-µm Pore Polycarbonate Membrane Inserts (Corning). First, transwells were settled onto 24-well plates (Corning) and coated with 50 µl of Matrigel matrix diluted in DMEM (1:3) and incubated at 37°C for 1 h. CRC cells were harvested with Trypsin-EDTA (Lonza), and 1 × 10^6^ cells were resuspended in 100 µl sterile adhesion medium and transferred to a precoated transwell. One milliliter of 10% FBS DMEM was added to each well as chemoattractant. At this point, cells were treated with or without 5 or 10 mM of NAC and 5 or 10 mM of metformin. Subsequently, plates were incubated for 22 h at 37°C and 5% CO_2_. Then, non-invaded cells and Matrigel were removed from the upper membrane surface of the transwells with cotton swabs, and invaded cells on the lower membrane surface were fixed by adding 2 ml of 4% paraformaldehyde to each well for 1 h at room temperature (RT). Next, transwells were transferred to new 24-well plates and stained with 2 ml of 0.2% crystal violet and 25% methanol for 30 min at RT. Finally, transwells were washed with H_2_Omq to remove dye traces and photographed with the DMi1 Microscope (Leica), and cells were counted with the ImageJ program (Fiji).

### Protein Extract and Western Blot

Protein expression was analyzed by Coomassie Blue staining and Western blot (WB). First, CRC cells were harvested with Trypsin-EDTA (Lonza). Cells were lysed in 500 µl of lysis buffer (RIPA, Sigma-Aldrich) supplemented with 1× protease and phosphatase inhibitors (MedChemExpress, Princeton, NJ, USA) by mechanical disaggregation using 16- and 18-G needle syringes. Then, the protein extracts were centrifuged for 10 min at 4°C at 10,000 g, and supernatants were transferred to new tubes. The protein concentration was determined by tryptophan quantification (22, 26, 27) and confirmed by Coomassie blue staining. Five micrograms of each protein extract was separated by 10% sodium dodecyl sulphate-polyacrylamide gel electrophoresis (SDS-PAGE) under reducing conditions and transferred to nitrocellulose membranes. Membranes were then blocked with 0.1% Tween PBS supplemented with 3% skimmed milk for 1 h at room temperature. Subsequently, membranes were incubated with a mouse monoclonal anti-LDLR (1/2,000, Elabscience, Houston, TX, USA, E-AB-27729), a rabbit polyclonal anti-CD36 (1/1,000, NeoBiotech, Las Vegas, NV, USA, NB-22-37760), a rabbit polyclonal anti-FASN (1/1,000, Elabscience, E-AB-31416), and a rabbit polyclonal anti-GAPDH (1/2,000, Rockland, Pottstown, PA, USA, 800-656-7625) O/N at 4°C. The next day, membranes were washed three times with 0.1% Tween PBS and incubated with the secondary antibody HRP-conjugated goat anti-rabbit IgG (GAR, 1/3,000, Sigma-Aldrich) or HRP-conjugated goat anti-mouse IgG (GAM, 1/3,000, Sigma-Aldrich) for 1 h at room temperature. Finally, the membranes were washed three times with 0.1% Tween PBS and the signal developed using the ECL Pico Plus Chemiluminescent Reagent (Thermo Fisher Scientific, Waltham, MA, USA).

### *In Silico* Analysis of Prognostic Value

The COAD (colon adenocarcinoma) TCGA dataset was used to analyse the prognostic value of LDLR, CD36, FABP4, FASN, FABP1, APOA1, ABCA1, AGPAT1, SCD, LPL, and SREBF1. The prognostic value of these genes was evaluated by Kaplan–Meier curves using the median as the best cutoff to separate high- and low-expression populations. The significance of the difference in survival between both populations was estimated using the log-rank test. The GSE68468 database with transcript data from patients with primary colon cancer was used to analyse the association of these genes with metastasis using 47 metastatic samples to the liver, 20 metastatic samples to the lung, 186 CRC tumour samples, and 55 normal colon samples. Data were normalized using Bioconductor’s Affymetrix package and transformed into z-scores. Data and p values were calculated with ggplot2. For a proper visualization of the data, box-plots were made with GraphPad Prism 8.

### Analysis of Cell Bioenergetics

The oxygen consumption rate (OCR) and extracellular acidification rate (ECAR) were monitored to quantify the oxidative phosphorylation and aerobic glycolysis, respectively, using the extracellular flux bioanalyzer Seahorse (Seahorse Biosciences, North Billerica, MA, USA).

Prior to experiments, cell density and drug concentration were optimized. For the Mito Stress test, cells were seeded in DMEM 10% FBS, and the next day, the media were changed to 10 mM glucose, 2 mM glutamine, and 2 mM pyruvate in non-buffered DMEM base media adjusted to pH 7.4. Cells were then kept for 1 h in an incubator at 37°C, without CO_2_. The assay was performed as previously described (25), with minor modifications. In brief, basal OCR was first measured, and then different modulators of the respiration chain complexes were injected, following the specifications of the Mito Stress kit (2 μM oligomycin, 0.8 μM FCCP, and 0.5 μM rotenone/antimycin A). For Glyco Stress analysis, cells were seeded in DMEM base media in the absence of glucose with 1 mM pyruvate and 2 mM glutamine. Following the specifications of the Glycolysis Stress kit, 10 mM glucose was injected first, then 0.5 μM oligomycin, and finally, 50 mM 2-deoxy-D-glucose (2-DG). OCR and ECAR were measured three times after injection of each drug. Three independent experiments were performed, with six replicates per condition.

Alternatively, experiments were performed in the presence or absence of plasma from individuals with morbid obesity (body mass index, BMI >30 kg/m^2^—obese) and individuals with normal weight (BMI <25 kg/m^2^—NW) as a control of the assay. To this end, media were supplemented with 5% of plasma from these individuals instead of 10% FBS, as above indicated, in KM12C, KM12SM, and KM12L4a cells.

### Plasma From Volunteers

Plasma samples used in this study were provided by the Platform for Clinical Trials in Nutrition and Health (GENYAL) of the IMDEA Food Institute (Madrid, Spain). Informed consent was obtained from the subjects to conserve their sample for scientific studies within the line of research in nutrition and health. This informed consent was included in the IMD PI:030 clinical trial protocol approved by the IMDEA Food ethics committee.

Eight plasma samples were divided in two groups: A, plasma of volunteers with a body mass index (BMI) <25 kg/m^2^ (normal-weight NW), and group B, with a BMI >30 kg/m^2^ (Obese OB) (**Table S3**). The main parameters considered from anthropometric and biochemical data were sex, BMI, %total fat, %muscle mass, visceral fat classification, waist contour, systolic blood pressure, diastolic blood pressure, heart rate, total cholesterol, HDL cholesterol, LDL cholesterol, triglycerides, creatinine, liver health-related enzymes (aspartate aminotransferase, alanine aminotransferase), and ultrasensitive CRP.

### Transient CD36 Silencing

For transient CD36 silencing, transfection was performed in six-well plates using the jetPRIME reagent (PolyPlus Transfection) with, alternatively, MISSION esiRNAs targeting CD36 (EHU089321; Sigma-Aldrich) or control siRNAs (SIC001; Sigma-Aldrich) following the manufacturer’s instructions. Briefly, 2.5 × 10^5^ cells were transfected with 22 pmol siRNA using 2 µl of jetPRIME transfection reagent and 100 µl of jetPRIME buffer. Then, 48 h after transfection, cells were analyzed by semiquantitative PCR or Western blot (WB). Alternatively, transfected cells were used for cell-based assays as above or for *in vivo* analysis.

### *In Vivo* Animal Experiments

The Ethical Committee of the Instituto de Salud Carlos III (Spain) approved the protocols used for the experimental work with mice after approval by the OEBA ethical committee (Proex 285/19).

For marker analysis in tumour metastasis, 1 × 10^6^ KM12SM or KM12L4a cells were injected intrasplenically in nude mice (Charles River n = 3) in 0.1 ml PBS and distal metastasis collected at endpoint. For liver homing analysis, nude mice were inoculated intrasplenically with 1 × 10^6^ KM12SM or KM12L4a cells after 24 h of transient transfection with CD36 or control siRNAs (n = 2) in 0.1 ml PBS. Mice were euthanized 24 h after intrasplenical cell inoculation, and RNA from the liver, lung, and spleen was isolated using TRIzol Reagent. RNA was analyzed by RT-PCR to amplify human GAPDH and murine β-actin as loading control using specific primers (**Table S2**).

### Statistical Analysis

Significance between groups was determined by Student’s t-test. All reported p values were two-sided. Statistical significance was defined as p < 0.05. The statistical analyses were done with GraphPad Prism8.

## Results

### Meta-Analysis of Proteomics Data of KM12SM and KM12C Cell Lines Reveal Metabolic and EMT Differences Associated With Progression and Metastasis

An in-depth quantitative SILAC proteomics analysis of KM12SM in comparison to KM12C cells was previously performed (22, 23). Secretome protein analysis was subsequently followed by the subcellular fractionation of these cells to spatially analyse cytoplasmic, membrane, nuclear, chromatin-bound, and cytoskeletal proteins (22, 23). In these reports, dysregulated proteins in metastatic to liver CRC cells were quantified and their localization mapped. The data highlighted the importance of protein localization to distinguish proteins and complexes that behave differently in various organelles and locations to identify underlying mechanisms of CRC metastasis to the liver.

**Figure 1:**
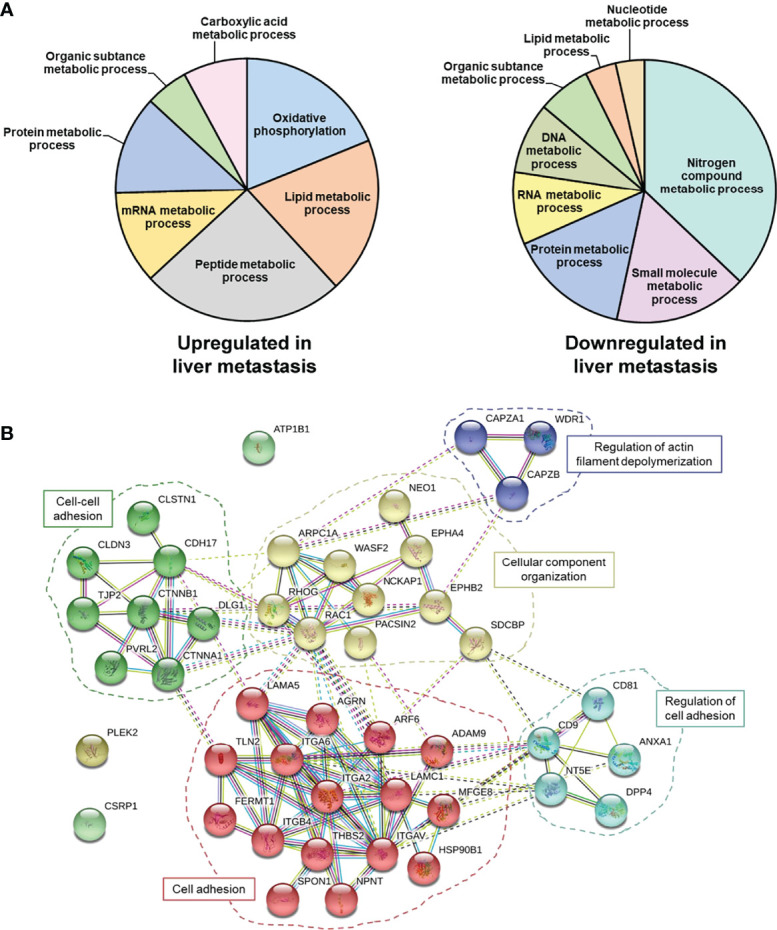
Meta-analysis of proteomics data of liver metastatic KM12SM in comparison to parental non-metastatic KM12C CRC cells. **(A)** Metabolic processes altered in liver metastasis of CRC cells. A total of 868 dysregulated proteins 334 upregulated and 534 downregulated—in KM12SM cells in comparison to KM12C CRC cells were associated with metabolism. **(B)** Forty-five dysregulated proteins related to EMT and stemness in CRC liver metastasis were also involved in processes related to cell-cell contacts.

Here, we first surveyed these proteomic data for the identification of protein alterations related to metabolism, EMT, and/or stemness (**Figure 1**), which would support increased plasticity and stemness of metastatic cells to facilitate metastasis and further colonization. Interestingly, gene ontology annotation of proteins altered between KM12C and KM12SM cells revealed 868 dysregulated proteins related to metabolism, with 334 of them differentially upregulated in KM12SM metastatic to liver cells (**Figure 1A** and **Table S1A**). These proteomic alterations affected several cellular metabolic processes. Among the altered pathways, we found oxidative phosphorylation and lipid- and protein-related metabolic processes, which were mostly upregulated in liver metastasis. On the other hand, metabolic pathways of nitrogen compounds and small molecules were mainly downregulated in liver metastasis (**Figure 1A** and **Figure S1**). In addition, we found 45 dysregulated proteins related to EMT and stemness such as Cadherin-17, metalloproteases, integrins, or proteins related to actin cytoskeleton. Most of these proteins, mainly integrins, cytokines, and chemokines, were involved in the remodelling of cytoskeleton and the extracellular matrix, as well as in the configuration of the local tumour and the premestastatic tumour microenvironment (**Table S1B** and **Figure 1B**).

Several studies have already described an association between alterations in metabolism and bioenergetics and the appearance of intrinsic protumorigenic features (12, 12, 16, 17). Hence, we hypothesized that the metabolic alterations along with additional changes in the proteome may coordinate signal cascades to sustain cell proliferation, survival, chemoresistance, and metastatic formation and thus might have prognostic potential for CRC patients. Furthermore, these mediators may target different cellular components in the tumour microenvironment including adipocytes, fibroblasts, and endothelial and immune cells, which can also help define specific organs of dissemination.

Then, to get further insights into metabolic differences associated with the organotropic dissemination, we interrogated the well-established KM12 cell model system of CRC metastasis by means of cell-based *in vitro* functional experiments, qPCR, WB analysis, extracellular flux bioenergetic analysis, and *in vivo* assays. Lipid metabolic specificities have been identified associated with their differential metastatic organotropism, and validated *in vitro* and *in vivo*. Importantly, these differences may constitute potential prognostic biomarkers in CRC patients as revealed by meta-analysis on the COAD TCGA database and help dictate metastatic foci as revealed by meta-analysis on the GSE68468 database containing information regarding RNA expression data of CRC patients (primary colon cancer, metastases, and matched normal mucosa).

### KM12SM and KM12L4a CRC Cells Display Increased Invasiveness *In Vitro* Compared to Their Control Isogenic Parental KM12C Cells

The EMT program confers critical properties for proliferation, adhesion, extracellular matrix remodelling, invasion, and metastatic dissemination (28).

**Figure 2:**
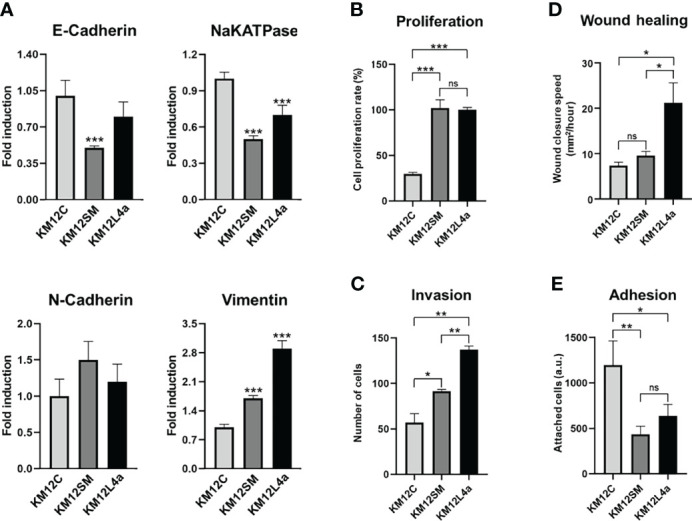
Characterization of metastatic KM12SM and KM12L4a CRC cells in comparison to the isogenic non-metastatic KM12C CRC cells. **(A)** qPCR analysis showed the upregulation of mesenchymal markers (N-cadherin and vimentin) and the downregulation of epithelial markers (E-cadherin and NaKATPase) in both metastatic CRC cells compared to KM12C cells. **(B–E)** *In vitro* functional cell-based assays showed that metastatic CRC cells possessed increased tumorigenic and metastatic properties than non-metastatic KM12C CRC cells. **(B)** Proliferation. **(C)** Invasion. **(D)** Wound healing. **(E)** Adhesion. *p value < 0.05; **p value < 0.01; ***p value < 0.001.

First, we corroborated by qPCR the statistically significant dysregulation of EMT markers, *E-cadherin*, *NaKATPase*, and *Vimentin*, in the KM12 cell system. Furthermore, although not significant, the opposite dysregulation of *N-cadherin* in the KM12 cell system in comparison to *E-cadherin* was also observed. These results confirmed the upregulation of mesenchymal markers and the downregulation of epithelial markers in both metastatic CRC cells compared to KM12C parental cells (**Figure 2A**). The initial acquisition of an EMT phenotype in the primary tumour and the reversion to a MET phenotype in secondary niches are crucial for the establishment of metastasis and cancer progression.

As illustrated in **Figures 2B–E**, KM12SM and KM12L4a cells showed increased tumorigenic and metastatic properties in comparison to non-metastatic KM12C CRC cells. KM12L4a and KM12SM metastatic CRC cell lines proliferated (**Figure 2B**) and invaded through Matrigel-coated chambers (**Figure 2C**) to a higher extent than the parental KM12C cell line. Furthermore, KM12SM and KM12L4a showed higher migratory capacity (wound closure speed) than KM12C control cells, with KM12L4a showing statistically higher migratory capacity than control and KM12SM cells (**Figure 2D**). In contrast, the adhesion abilities of KM12L4a and KM12SM highly metastatic cells were reduced compared to KM12C cells, supporting their increased ability for cell dissemination (**Figure 2E**). Moreover, the analysis of stemness markers showed increased phenotypic plasticity in metastatic KM12SM and KM12L4a cells, supporting not only dissemination but also the potential for subsequent metastatic colonization (**Figure S2**).

Collectively, these data validated our previous findings showing dysregulation in EMT and stemness in highly metastatic CRC cells.

### Metastatic KM12SM and KM12L4a CRC Cells Have Higher Levels of SO* and Ψm at Mitochondria

In carcinoma cells, the EMT program can be triggered by heterotypic signals, such as somatic mutations sustained during primary tumour formation, intracellular and extracellular signalling pathways, and even signals from the tumour-associated stroma.

**Figure 3:**
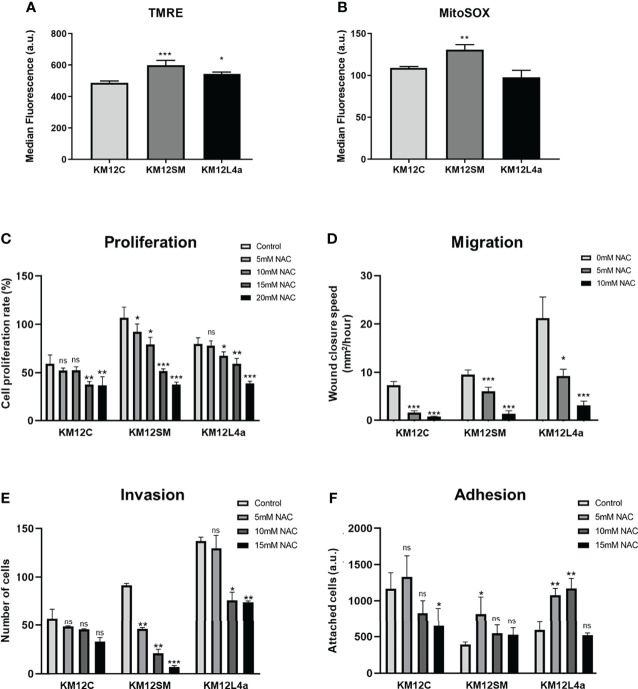
Reactive oxygen species effects on CRC cells. **(A, B)** Quantification by FACS analysis of Ψm at mitochondria and ROS species. Statistical analysis is referred to the comparisons with KM12C cells. **(A)** A TMRM probe was used to quantify the mitochondrial membrane potential (Ψm). **(B)** A MitoSOX-Red probe was used to determine the levels of mitochondrial superoxide anions (SO^*^). Analysis of the effect of NAC (0–20 mM) on proliferation **(C)**, migration **(D)**, invasion **(E)**, and adhesion **(F)** ability of metastatic and non-metastatic KM12 CRC cells. Wound closure speed is referred to wound size differences between 0 and 62 h of each experiment. Metastatic CRC cells were more dependent on the high levels of ROS to maintain their properties, with KM12SM cells being the most dependent of ROS. **(C–F)** Statistical analysis is referred to the cells without NAC treatment. *p value < 0.05; **p value < 0.01; ***p value < 0.001.

Increased levels of reactive oxygen species (ROS) have been shown to promote survival and dissemination of cancer cells (13). ROS produced as by-products of metabolism have been shown to play a critical role in cancer initiation and progression (29). For this reason, we interrogated the KM12 CRC cell system for the levels of mitochondrial membrane potential (Ψm)—TMRE staining—and the levels of the mitochondrial derived superoxide anions (SO*)—MitoSOX staining. We found that KM12SM and KM12L4a metastatic cells showed increased levels of Ψm, suggesting a mitochondrial switch favouring anabolism, in line with the increased cell proliferation compared to KM12C control cells (**Figure 3A**). Interestingly, only KM12SM cells displayed increased levels of SO* produced in mitochondria compared to control cells (**Figure 3B**). These results indicate that metastatic CRC cells have differences in the performance of the oxidative phosphorylation in mitochondria.

To further confirm these results, we analysed the effect of N-acetylcysteine (NAC) on cell proliferation, migration, invasion, and adhesion assays (**Figures 3C–F** and **Figure S3A**). NAC has been described as an exogenous antioxidant that mimics the effects of natural antioxidants. It is an aminothiol whose anti-ROS activity results from its free radical scavenging capacity, which originates from the redox potential of thiols and from the increase it induces in the cellular levels of cysteine and intracellular glutathione (GSH), a substrate of ROS scavenging enzymes (30, 31). Analysis of the tumorigenic capacities of high and low metastatic KM12 cells in the presence of different amounts of NAC revealed differences in metastatic KM12SM and KM12L4a cells at 5 and 10 mM of NAC treatment, whereas higher NAC concentrations induced cellular death. We observed that in the presence of NAC, the proliferation, migration, and invasion capacity of KM12SM and KM12L4a cells were statistically significant delayed, with KM12SM being the most affected cell line, while their adhesion capacity was increased. In contrast, higher concentrations of NAC (between 15 and 20 mM NAC) were needed to significantly affect the capabilities of KM12C cells. These results support the idea that KM12SM and KM12L4a cells require higher levels of ROS than KM12C cells to activate signalling pathways to support their tumorigenic abilities.

**Figure 4:**
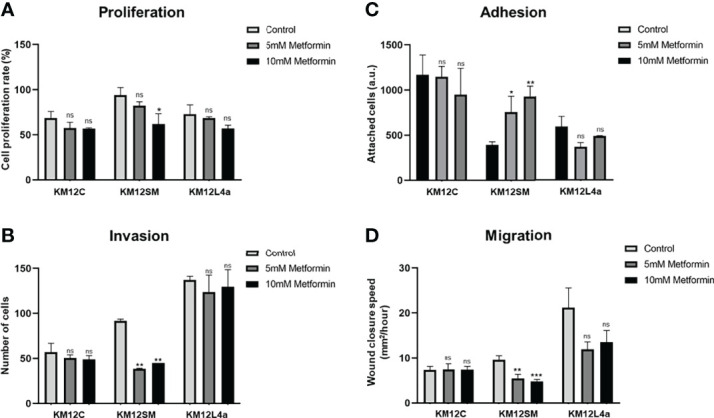
Analysis of the effect of metformin on the tumorigenic and metastatic properties of CRC isogenic cells with metastatic and non-metastatic properties. **(A)** Proliferation, **(B)** invasion, **(C)** adhesion, and **(D)** migration abilities of KM12 cells in the presence or absence of 10–15 mM metformin are represented. Wound closure speed is referred to wound size differences between 0 and 62 h of each experiment. KM12SM metastatic cells were the most affected by this complex I inhibitor compound, suggesting that these cells are not able to respond to increased cellular stress. *p value < 0.05; **p value < 0.01; ***p value < 0.001.

In addition, we also analysed the effects of metformin treatment by means of cell proliferation, invasion, adhesion, and wound healing assays (**Figure 4** and **Figure S3B**). Metformin disrupts mitochondrial respiration by interfering with mitochondrial complex I, which decreases ATP synthesis and activates AMPK, inducing cell metabolic stress and increasing the levels of intracellular ROS (32, 33). Here, we observed that this AMPK activator mainly delayed the proliferation and invasion capacity of KM12SM cells (**Figures 4A, B**), whereas KM12L4a and KM12C cells did not show significant differences in its presence or absence. In addition, the adhesion capacity of KM12SM cells was increased in the presence of metformin (**Figure 4C**), while the adhesion capacity of KM12L4a and KM12C cells was unaffected. On the other hand, metformin produced a significant decrease in the migration capacity of KM12L4a and KM12SM cells (**Figure 4D**), while the migration of KM12C cells remained unaltered. Remarkably, KM12L4a migrated 33% less than control cells without drugs, while KM12SM migratory capacity was reduced by more than 50%.

Collectively, these data show that metastatic KM12SM and KM12L4a cells are more dependent on intracellular ROS levels than non-metastatic KM12C cells, with KM12SM cells requiring the highest amount of ROS to maintain their metastatic properties. Furthermore, we observed that KM12SM cells were unable to respond to the increased cellular stress after metformin treatment compared to KM12L4a cells, highlighting the high dependence of KM12SM cells on oxidative phosphorylation.

### Cell Bioenergetics of Main Energetic Pathways

**Figure 5:**
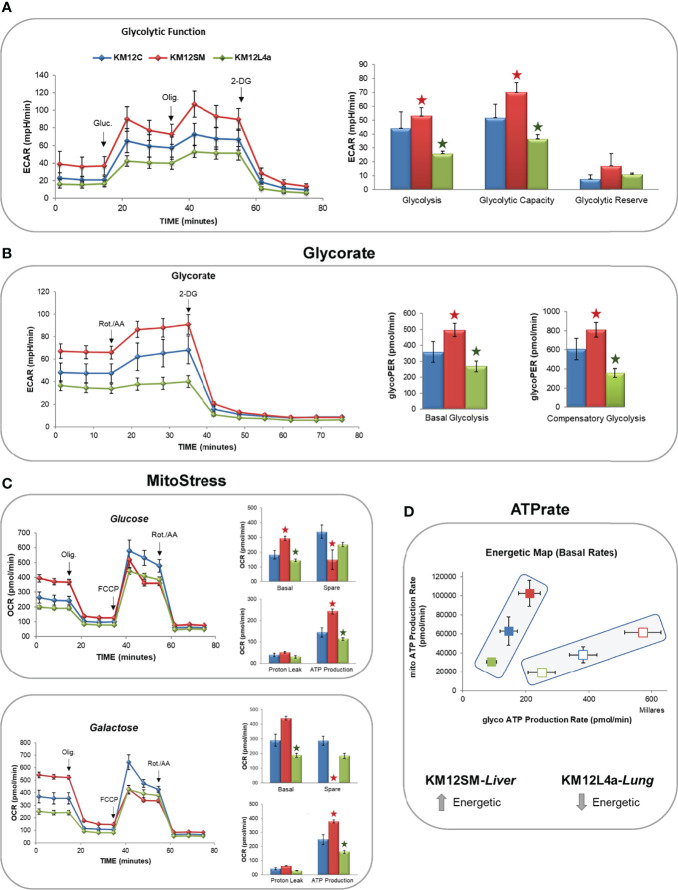
Monitorization by flux analysis of aerobic glycolysis and mitochondrial oxidative phosphorylation. **(A)** Analysis of ECAR glycolysis stress. Cells were incubated overnight in low-glucose media (5 mM glucose). The next day, the medium was changed to DMEM base media in the absence of glucose for 1 h, and the basal levels of ECAR were determined. After the injection of 10 mM glucose (Gluc), the increase in ECAR was used to quantify aerobic glycolysis. Next, after the injection of oligomycin, the increased ECAR allowed the determination of the maximal glycolytic capacity. **(B)** GlycoRate test. **(C)** Mito Stress test in the presence of glucose or galactose. **(D)** Basal OCR (BRR), ATP production, and maximal OCR are shown after the sequential injections of oligomycin (Olig.), FCCP, and rotenone/antimycin A (Rot./AA). 2-DG: 2-deoxy-D-glucose. *p value < 0.05.

Then, to gain insights into functional bioenergetics and determine whether metabolic differences in the metastatic CRC cells may contribute to their different metastatic tropism, we proceeded to analyse in detail the bioenergetic profile of KM12C, KM12SM, and KM12L4a cells. To this end, aerobic glycolysis and mitochondrial respiration were analysed by monitoring ECAR and OCR rates (**Figure 5**).

#### Aerobic Glycolysis

After 1 h of glucose starving, basal ECAR, which is an indirect readout of L-lactate production by aerobic glycolysis, was measured. Interestingly, basal ECAR of KM12SM cells was higher than that observed in parental cells. In contrast, KM12L4a cell basal ECAR was decreased compared to parental cells (**Figure 5A**). Next, we injected glucose into the media to determine the cells’ ability to increase the glycolytic pathway. KM12SM cells showed the highest response associated with increased levels of ECAR. In contrast, although capable of responding to glucose, KM12L4a cells showed reduced levels of ECAR compared to KM12C cells. Finally, KM12SM cells continued to increase glycolysis to a higher maximum ECAR than parental cells when oligomycin was injected to block ATP production from mitochondria. In contrast, the maximum ECAR for KM12L4a cells was the lowest compared to parental cells.

Then, to further confirm that the observed differences were due to changes in aerobic glycolysis, we analysed glycoPER. In this assay, cells were maintained in complete media (10 mM glucose, 1 mM pyruvate, and 2 mM glutamine) to apply an experimentally determined buffer (provided by Seahorse Bioscience) which allows to determine the contribution of H^+^ to the Proton Extrusion Rate (PE) from aerobic glycolysis (L-lactate and H^+^) or mitochondrial oxidative phosphorylation (CO_2_+H_2_0-HCO_3_^-^ +H^+^). As shown in **Figure 5B**, basal glycolysis was higher in KM12SM and lower in KM12L4a compared to control parental cells. Next, we injected rotenone/antimycin A to shut down mitochondrial complexes I and III and induce a compensatory upregulation of aerobic glycolysis. Furthermore, compensatory stressed glycolysis of KM12SM cells was higher than that of control cells. In contrast, KM12L4a cells showed the lowest levels of compensatory ECAR. Finally, we injected 2-deoxyglucose, a competitive inhibitor of glucokinase, to demonstrate that ECAR was associated with aerobic glycolysis.

These results clearly show that KM12SM and KM12L4a cells have different performances associated with aerobic glycolysis compared to control non-metastatic KM12C parental CRC cells.

#### Mitochondrial Oxidative Phosphorylation

Due to the differences observed independently of aerobic glycolysis, and the fact that KM12SM cells displayed increased levels of ROS species, we wanted to interrogate KM12 cells regarding mitochondrial oxidative phosphorylation (**Figure 5C**).

Therefore, OCR levels were measured after the sequential injection of drugs that modulate the oxidative phosphorylation in mitochondria. KM12SM cells showed the highest basal respiration rates (BRR) in comparison to KM12C cells. In contrast, KM12L4a cells showed the lowest BRR. When oligomycin (1 µM) was injected to inhibit the V-ATPase complex, the OCR levels of KM12SM cells were reduced to the OCR levels of KM12L4a and parental cells. Interestingly, after injection of FCCP (0.4 µM) to uncouple the electron transport chain (ETC) from ATP synthesis, the maximal respiration rate (MRR) of KM12SM cells was lower than that of KM12L4a and parental cells. These results suggest that the respiration rate of KM12SM cells is compromised under stress conditions, indicating that they are at their maximal respiratory capacity. This is in agreement with the higher levels of ROS and membrane potential at mitochondria (Ψm) observed in KM12SM cells. To confirm this result, we conducted a Mito Stress test by adding 10 mM galactose, which is a substrate that generates higher levels of ROS compared to glucose. All cell lines increased their basal respiration rate compared to that observed in the glucose condition. Again, KM12SM showed the highest BRR and KM12L4a the lowest BRR. Interestingly, KM12SM cells showed similar rates of basal and maximal respiration, suggesting that they are highly oxidative cells under basal conditions and that this oxidative capacity cannot be augmented under stress conditions. In contrast, KM12L4a cells showed the lowest levels of both BRR and MRR. In conclusion, these results indicate that KM12SM cells are energetically very active, while KM12L4a are the least energetic cells.

#### ATP Rate and Energetic Phenotype

Finally, ATP rate analysis of KM12SM and KM12L4a cells showed interesting bioenergetic differences among KM12 cells. While parental isogenic non-metastatic KM12C cells showed an intermediate cell bioenergetics, liver metastatic KM12SM cells showed the highest ATP production under basal or stressed conditions. In contrast, KM12L4a showed the lowest levels of ATP production (**Figure 5D**). These results are in agreement with the increased glycolytic and oxidative phosphorylation observed in KM12SM cells (the highest bioenergetic profile) and the lower bioenergetic profile of KM12L4a cells, both independent of aerobic glycolysis and oxidative phosphorylation.

### Metastatic KM12L4a CRC Cells Display Higher Dependency on Exogenous Fatty Acid Uptake

The mechanisms whereby some tumour cells detach from the primary lesion to colonize distant sites are still largely unknown, as well as pro-metastatic events occurring in the majority of solid tumours. As the proliferation rates in KM12SM and KM12L4a cell lines were similar, metabolic differences, regardless of cell bioenergetics, may contribute to differences in other cell signalling and protumorigenic features. Changes in lipid metabolism have been recognized as crucial players affecting cancer cell survival, progression, and therapy response. Furthermore, since tumour cells are often exposed to a metabolically challenging environment, mediators of lipid metabolism are key players in controlling the associated metabolic stress and in shaping the stroma and the cellular components of the tumour microenvironment.

**Figure 6:**
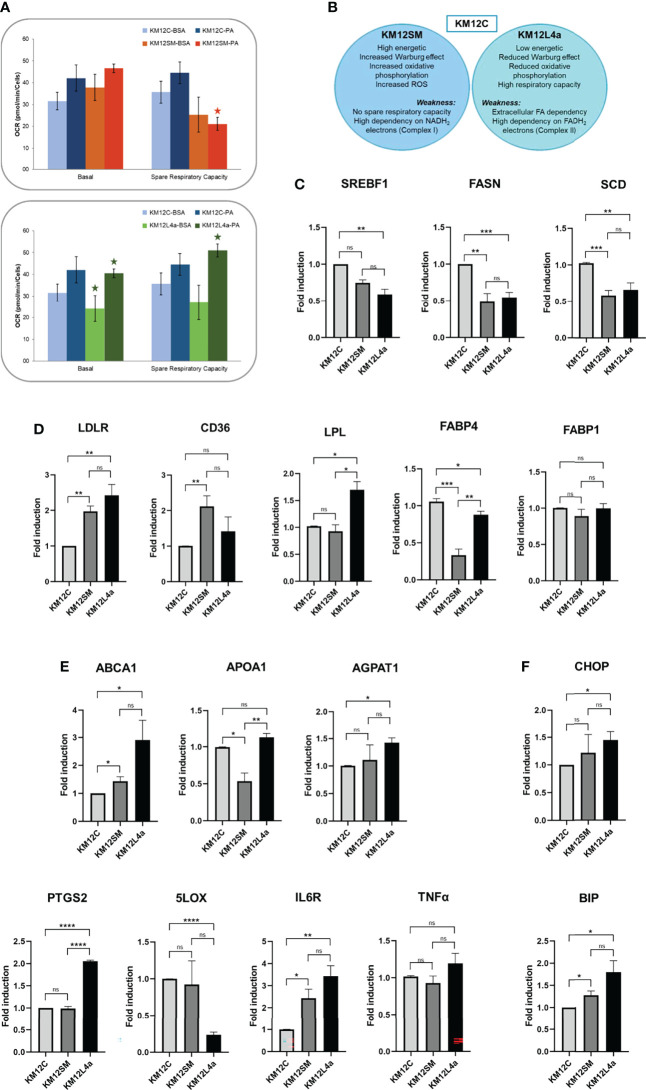
Analysis of the expression levels of lipid metabolism genes in KM12C and KM12SM and KM12L4a. **(A)** Exogenous uptake of FAs and cholesterol. The mitochondrial oxidative phosphorylation response to FA supplementation was analysed with the Seahorse bioanalyzer. **(B)** Schematic of the strengths and weaknesses of the metastatic isogenic KM12SM and KM12L4a cells as observed from the analyses. qPCR analysis of the expression levels of lipid metabolism genes related to **(C)** *de novo* synthesis of FA, **(D)** intracellular cholesterol homeostasis and plasmatic membrane lipid remodeling-related genes, **(E)** cholesterol and membrane lipid remodeling and of lipid metabolism-related inflammatory markers, and **(F)** cellular stress mediators in KM12SM and KM12L4a referring to KM12C parental cells. *p value < 0.05; **p value < 0.01; ***p value < 0.001; ****p value < 0.0001.

Then, to functionally confirm the dependence of metastatic CRC cells on exogenous fatty acid supply, we monitored, using the Seahorse bioanalyzer, the mitochondrial oxidative phosphorylation response to extracellular fatty acid (FA) supplementation. Briefly, after an O/N starvation (low glucose—0.5 mM glucose, 1% FBS, 0.5 mM carnitine), we challenged cells to a palmitic acid-BSA (PA) input and monitored OCR. Control-BSA was also included as a negative control. Interestingly, we found that KM12L4a and KM12SM cell lines only increased their basal OCR compared to control cells when PA was added, especially KM12L4a cells (**Figure 6A**). Furthermore, we observed that KM12SM exhibited a lower spare respiratory capacity after PA supplementation, being unable to respond to the additional energy demand under stress conditions (after FCCP injection), as previously shown in glucose enriched media. On the other hand, the spare respiratory capacity of KM12L4a cells was significantly increased with PA, compared to control-BSA, suggesting that KM12L4a cells are more dependent on the exogenous FA uptake to increase their cell bioenergetic capacity.

In summary, KM12SM cells showed an increased Warburg effect, increased oxidative phosphorylation, and increased ROS, and, as weakness, reduced spare respiratory capacity (in complete media, or in FA-supplemented media), related to a low capacity to respond to additional energy demand, while KM12L4a cells showed a reduced Warburg effect, reduced oxidative phosphorylation, high respiratory capacity, and, as weakness, high dependence on exogenous FA (**Figure 6B**).

Next, to further investigate the FA dependence of metastatic CRC cells compared to parental KM12C CRC cells, we analyzed the expression of genes related to lipid metabolism. We focused on genes involved in *de novo* fatty acid synthesis (*SREBF1*, *FASN*, *SCD*); exogenous lipid uptake (*LDLR*, *CD36*, *LPL*, *FABP1*, *FABP4*); remodelling of cholesterol and membrane lipids (*ABCA1*, *APOA1*, *AGPAT1*); and inflammation (*PTGS2*, 5*LOX*, *IL6R*, and *TNFA*), which could explain, at least partially, the observed differences in cell bioenergetics in KM12SM and KM12L4a cells compared to KM12C parental cells (**Figures 6C–E**). Strikingly, both metastatic CRC cells, KM12SM and KM12L4a, showed reduced expression levels of *SREBF1* (master transcription factor involved in *de novo* synthesis of fatty acids) and its downstream molecular targets *FASN* and *SCD* compared to control cells (**Figure 6C**). In contrast, metastatic KM12SM and KM12L4a CRC cells showed an increased expression of genes related to exogenous lipid uptake. More specifically, KM12L4a cells had increased gene expression levels of *LDLR*, *CD36*, and *LPL* and reduced gene expression levels of *FABP4* compared to KM12C cells, whereas KM12SM cells showed increased *LDLR* and *CD36* gene expression levels and reduced *FABP4* gene expression levels compared to parental KM12C control cells (**Figure 6D**).

Interestingly, the analysis of genes involved in plasma membrane lipid remodelling showed the upregulation of *ABCA1* and *AGPAT1* in KM12L4a cells compared to parental KM12C control cells, whereas KM12SM cells showed increased expression levels of *ABCA1* and reduced expression levels of *APOA1* compared to KM12C cells (**Figure 6E**). Furthermore, analysis of inflammatory lipid mediators also highlighted specific differences between the two metastatic CRC cell lines compared to KM12C cells. KM12L4a cells showed increased expression levels of *PTGS2* along with decreased 5*LOX*. Given that both enzymes have arachidonic acid (AA) as substrate, these results suggest that KM12L4a cells favour inflammatory mediators downstream of the prostaglandin pathway, as evidenced by the increase in the downstream mediator *IL6R*, which was also upregulated in KM12L4a cells (**Figure 6E**). In addition, although not significant, the increase of another downstream mediator (*TNFA*) was also observed, supporting the results observed for *IL6R* regarding inflammatory mediators.

Finally, *BIP* and *CHOP* were found upregulated in both metastatic cell lines, but the differences were statistically significant only in KM12SM cells, suggesting an increased metabolic stress in these cell lines (**Figure 6F**).

Collectively, the analysis of a panel of enzymes related to lipid metabolism has allowed for the identification of three metabolic axes associated with the differential organotropic dissemination involved in (i) the extracellular uptake of FAs and cholesterol vs. *de novo* lipogenesis, (ii) lipid-associated inflammatory networks, and (iii) lipid enzymes that affect plasma membrane remodelling, plasticity, and fluidity. Furthermore, these results highlight the role of *SREBF1*, *FASN*, *SCD*, *LDLR*, *CD36*, *FABP1*, *FABP4*, *LPL*, *PGCTS2*, *LOX5*, *IL6R*, *ABCA1*, *APOA1*, and *AGPAT1*, as differential lipid metabolism biomarkers of lung or liver tropism.

### Effect of Exogenous Fatty Acids From Plasma of Morbid Obesity and Normal-Weight Individuals on the KM12 Cell System

Several retrospective studies analysing large cohorts of CRC patients highlight the impact of obesity on overall survival (34). In addition, the mortality of CRC patients with a BMI between 25 and 50 kg/m^2^ increases linearly, while in patients with a BMI between 15 and 25 kg/m^2^ the risk of mortality does not vary.

Given that lipid metabolism targets related to extracellular lipid uptake were found to be upregulated in metastatic KM12SM and KM12L4a cells compared to KM12C control cells, we next wanted to evaluate the effect of obesity on the biological behaviour of the tumoural cells. Since obesity is characterized by increased levels of circulating free fatty acids that promote low-grade chronic inflammation (35), we hypothesized that incubation of KM12 cells with plasma from volunteers with a BMI <25 (group A: NW), or volunteers with a BMI >30 (group B: OB), would affect the behaviour of the cells differently.

**Figure 7:**
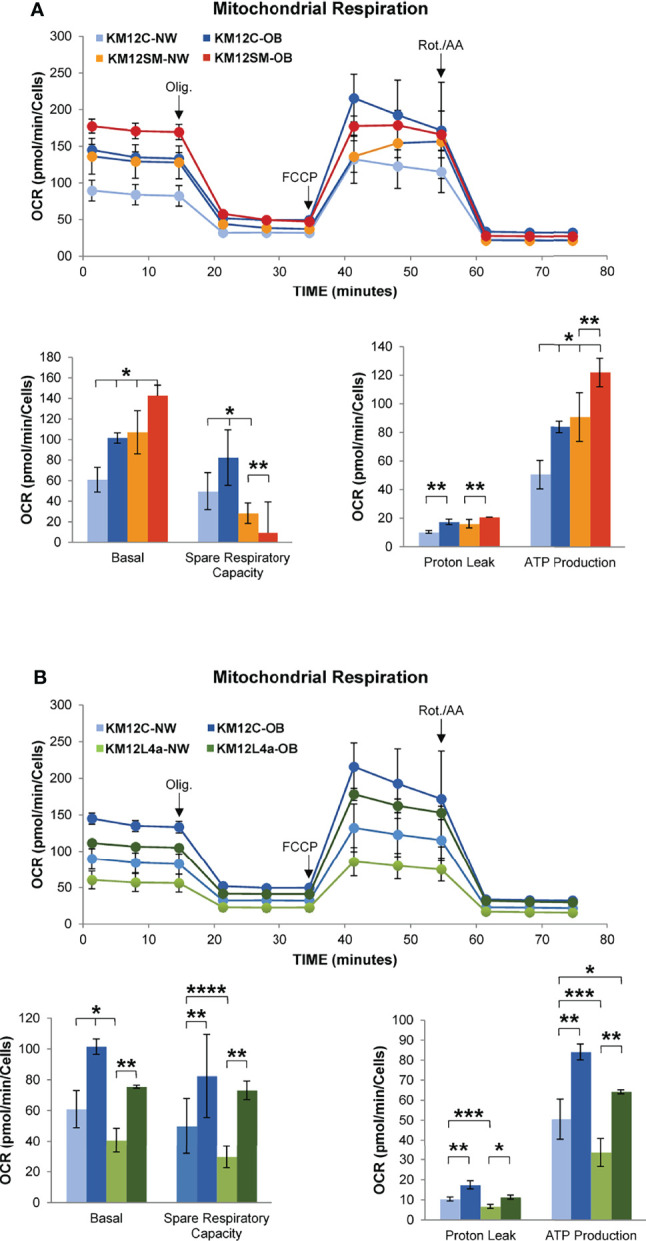
Investigation by flux analysis of mitochondrial respiration of KM12 cells incubated with plasma from volunteers. **(A)** Analysis of mitochondrial respiration of KM12C and KM12SM cells incubated with 5% plasma of volunteers with BMI <25 (NW) or BMI >30 (OB). Basal OCR and spare respiratory capacity, proton leak, and ATP production are shown. **(B)** Analysis of mitochondrial respiration of KM12C and KM12L4a cells incubated with 5% plasma of volunteers with BMI <25 (NP) or BMI >30 (OB). Basal OCR and spare respiratory capacity, proton leak, and ATP production are shown. Olig, oligomycin; Rot./AA, rotenone/antimycin **(A)** *p value < 0.05; **p value < 0.01; ***p value < 0.001; ****p value < 0.0001.

The supplementation with 5% of plasma from OB volunteers significantly enhanced the basal respiration and the ATP production of KM12C cells compared to that in the presence of plasma from NW volunteers, suggesting that plasma from OB volunteers is enriched on FAs and/or growth factors. In a similar way, plasma from OB volunteers significantly enhanced the basal respiration and the ATP production of KM12SM cells compared to that in the presence of plasma from NW volunteers but not their spare respiratory capacity (**Figure 7A**). Importantly, supplementation with 5% of plasma from OB volunteers significantly enhanced the basal and spare respiratory capacity and the ATP production of KM12L4a cells compared to that of supplementation with plasma from NW volunteers (**Figure 7B**). Furthermore, the data collected with NW plasma were in agreement with the response obtained at the basal state (**Figure 5C**).

These data indicate that obesity may stimulate cell migration capacity, stemness, and/or angiogenesis. Furthermore, obesity can remodel the tumour microenvironment leading to a chronic inflammatory state to stimulate dissemination, which will decrease the overall survival of CRC patients (36).

### Analysis of CD36 Lipid Metabolism Biomarker in Lung and Liver Tropism

Next, the ability of the KM12 CRC cell system to take up extracellular fatty acids was blocked by siRNAs to CD36 to determine whether it would have any effect on their metastatic ability, and to analyse whether the markers could be functionally important for the metastatic phenotypes.

**Figure 8:**
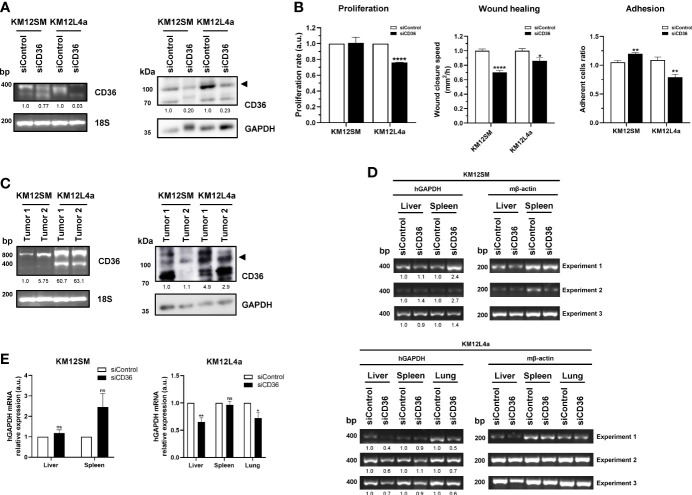
*In vitro* and *in vivo* effect of CD36 depletion in the tumorigenic and metastatic properties of KM12SM and KM12L4a cells or derived tumours. **(A)** Evaluation by PCR and WB of the transient silencing of CD36 after 48 h post-transfection in KM12SM and KM12L4a cell lines. As control, we transfected the cell lines with a scramble siRNA. By PCR, 18S was used as internal control. By WB, GAPDH was used as loading control. **(B)** Proliferation, wound healing, and adhesion properties of KM12SM and KM12L4a transiently transfected with CD36 siRNA in comparison to scramble siRNA were investigated, showing that CD36 plays a role in the tumorigenic and metastatic properties of metastatic colorectal cancer cell lines. **(C)** Analysis of the CD36 mRNA and protein expression levels in actual metastatic tumoral samples (n = 2) derived from KM12SM and KM12L4a cells. By PCR, 18S was used as internal control. By WB, GAPDH was used as loading control. **(D)** Nude mice intrasplenically inoculated with KM12SM or KM12L4a cells transiently transfected with siScramble and CD36 siRNA were sacrificed 24 h after inoculation for analysis of *in vivo* liver and lung homing (n = 3). RNA was isolated from the liver, lung, and spleen (as control) and directly subjected to RT-PCR to amplify human GAPDH (hGAPDH). Murine β-actin (mβ-actin) was amplified as control. **(E)** Statistically significant lower mRNA levels of hGAPDH were found for liver and lung homing of KM12L4a cells. **(A–D)** mRNA or protein abundance was quantified by densitometry using ImageJ, and data were normalized with the expression levels of 18S or mβ-actin for mRNA and GAPDH for protein abundance. *p value < 0.05; **p value < 0.01; ****p value < 0.0001.

Transient CD36 silencing followed by adhesion, proliferation, and wound healing assays was performed on KM12SM and KM12L4a cells compared to scrambled cells to assess the influence of CD36 on the tumorigenic and metastatic properties of cells. First, CD36 depletion by transient silencing was efficiently achieved as observed by PCR and WB analyses (**Figure 8A**). In proliferation assays, CD36-depleted KM12L4a cells proliferated less than control cells transfected with the scrambled siRNA (*p* value < 0.05), while KM12SM cells were mostly unaffected (**Figure 8B**). Wound healing assays were then used to analyze the role of CD36 in cell migration. Both CD36-silenced cell lines closed the wound at a slower rate than their control cells transfected with scramble siRNA with a significantly higher effect on KM12SM cells. Regarding the effect of CD36 on adhesive properties, cells transfected with CD36 siRNA showed opposite properties. KM12SM upon CD36 depletion showed a significant 20% higher adhesion than control cells, while KM12L4a cells showed a significant 32% lower adhesion ability to Matrigel than control cells.

These results suggest that CD36 silencing preferentially affects KM12L4a cells to a greater extent than KM12SM cells. For further confirmation, *in vivo* experiments were performed. First, the differential expression of CD36 was analyzed by PCR and WB in samples of metastatic tumours derived from intrasplenic injection of KM12SM and KM12L4a cells (**Figure 8C**). Metastatic tissue showed an enhanced CD36 expression in KM12L4a cell-derived metastatic tumour than in KM12SM cell-derived metastatic tumour, which would support the higher significant changes in the *in vitro* cell-based assays for KM12L4a cells than for KM12SM cells. Next, the *in vivo* effects on lung and liver homing of transient CD36 depletion were investigated in KM12SM and KM12L4a CRC metastatic cells. KM12SM and KM12L4a cells were inoculated into the spleens of nude mice to examine the effect of CD36 on the capacity for liver and lung homing. As a surrogate marker for homing, human GAPDH was detected by PCR, using mβ-actin as a control. Scramble and CD36 siRNA-transfected KM12SM cells were detected at similar extents in the liver, suggesting that CD36 had no effect on the homing of KM12SM cells. However, KM12L4a cells were significantly detected at a lower extent in the liver and lung upon CD36 depletion in comparison to control cells transfected with scramble siRNA (**Figures 8D, E**).

Collectively, as the bioenergetics-derived results suggested, CD36 depletion preferentially impaired homing produced by KM12L4a cells, whereas liver homing was unaffected by CD36-deficient KM12SM cells.

### Exogenous FA Transporters as Prognostic Markers of CRC

**Figure 9:**
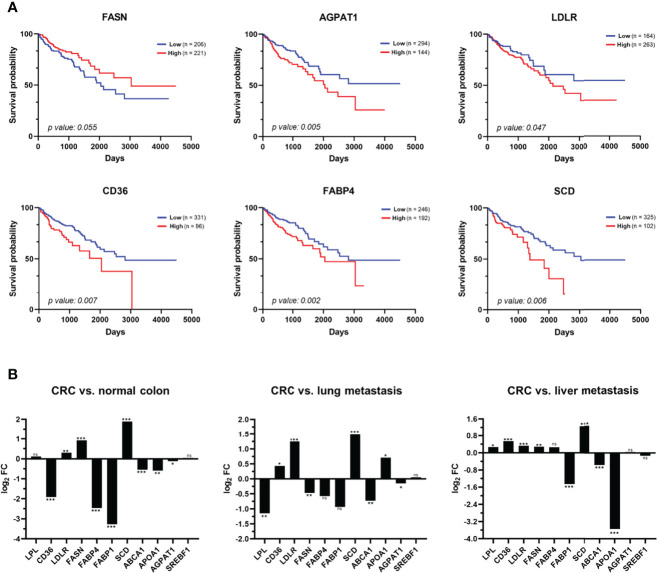
Analysis of the clinical relevance of the metabolic alterations in CRC patients. **(A)** Meta-analysis of mRNA levels of *FASN*, *AGPAT1*, *LDLR*, *CD36*, *FABP4*, and *SCD* in tissue samples of CRC patients using TCGA data related to colon adenocarcinoma (COAD). Kaplan–Meier analyses of overall survival of patients with colon cancer showed that a high expression of *AGPAT1*, *LDLR*, *CD36*, *FABP4*, and *SCD* are prognostic factors of worst prognosis for CRC patients. **(B)** The dataset from the NCBI Gene Expression Omnibus GSE68468 was used to analyse 47 metastatic samples to the liver and 20 metastatic samples to the lung from patients with primary colon cancer in comparison to 186 CRC primary tumour samples and 55 normal colon samples. Box-plots in log_2_ revealed a dysregulation of nine of the 11 proteins analysed in CRC primary tumour in comparison with normal colon mucosa (left panel). In addition, a statistically significant upregulation of *LPL*, *FASN*, *AGPAT1*, and *ABCA1* in lung metastasis in comparison with primary CRC tumour (middle panel) was found, whereas *FABP1*, *ABCA1*, and *APOA1* were found statistically upregulated in liver metastasis in comparison with primary CRC tumour (right panel). FC, fold change; ns, not-significant; *p value < 0.05; **p value < 0.01; ***p value < 0.001.

Finally, we hypothesized that these findings may be reflected in actual CRC samples from patients. To address this question, we analysed the mRNA levels of the lipid metabolism biomarkers *SREBF1*, *FASN*, *SCD*, *LDLR*, *CD36*, *LPL*, *FABP1*, *FABP4*, *ABCA1*, *APOA1*, and *AGPAT1* in actual tumoural tissue samples by meta-analysis of the COAD TCGA dataset, to determine their usefulness as prognostic markers. Additionally, we used the GSE68468 database to analyse their association to different CRC metastatic foci (**Figure 9**).

Remarkably, we observed statistically significant differences in patient survival according to *FASN*, *AGPAT1*, *LDLR*, *CD36*, *FABP4*, and *SCD* expression levels (**Figure 9A**). The 5-year survival rate decreases to 60% with a high expression of *LDLR*, while it increases to 73% with a low expression of this receptor (*p* value < 0.05). Similarly, high expression levels of *CD36*, *FABP4*, *SCD*, and *AGPAT1* decrease this rate to 49%–55%, while the rate increases to 67%–71% with their low expression (*p* value < 0.05). On the other hand, a low expression of *FASN* decreases the 5-year survival rate to 60%, while it increases to 70% with its high expression (*p* value < 0.05). In conclusion, the expression levels of these proteins are prognostic factors of colon cancer, which could be associated with a more or less aggressive phenotype of CRC (**Figure 9A**).

Then, we analysed their potential association with metastatic foci. First, nine out of the 11 genes analysed were found statistically dysregulated in CRC in comparison to normal colon mucosa (**Figure 9B**). Furthermore, *ABCA1* and *FABP1* were found to be statistically upregulated in liver and lung metastasis compared to CRC tumour, while *SCD*, *CD36*, and *LDLR* were downregulated in both CRC metastastic foci (**Figure 9B**). Interestingly, *APOA1*, *FASN*, *FABP4*, and *LPL* were oppositely dysregulated in liver and lung metastasis compared to the primary CRC tumour, whereas *AGPAT1* was found to be significantly upregulated in lung metastasis (**Figure 9B**). Finally, the expression levels of these genes were compared between lung CRC metastasis and liver CRC metastasis. We found an upregulation of *LPL*, *CD36*, *LDLR*, *FASN*, *FABP4*, *SCD*, *ABCA1*, and *AGPAT1* and a downregulation of *FABP1*, *APOA1*, and *SREBF1* in lung CRC metastasis compared with liver CRC metastasis (**Figure S4A**). Remarkably, the protein expression levels of *CD36*, *LDLR*, and *FASN* in KM12SM and KM12L4a cells with different metastatic abilities were in agreement with data from CRC patients from the GSE68468 database (**Figure S4B**). These results further support the usefulness of the CRC metastasis KM12 cell system, which recapitulates quite effectively critical issues of CRC metastasis.

Collectively, these data indicate that the metabolic alterations found in isogenic CRC cells with different metastatic organotropisms partially resemble alterations in CRC patients that are associated with prognosis. The concordance observed between the expression levels of the indicated genes associated with organotropism of CRC cells and their expression in metastatic tissue of CRC patients suggests that these metabolic alterations—among others—help to dictate metastatic tropisms. Specifically, we have demonstrated here the role of *CD36*, *FASN*, *LDLR*, *FABP4*, *LPL*, *SCD*, and *APOA1* as markers of metastatic CRC tropism and validated *in vivo* the association of CD36 with the lung tropism of CRC cells.

## Discussion

It is well known that oncogenic transformation alters tumour metabolism to sustain cell growth and dissemination. Furthermore, cancer cells need to control metabolic stress to avoid cell death by activating pro-survival pathways (37). Moreover, by-products of cancer metabolism, such as ROS production, may support the EMT program to promote dissemination (13, 14). Thus, the exposition to matrix metalloproteinases (MMPs) (38), local inflammation (39), and aerobic glycolysis metabolites have been shown to activate the EMT program (15). In addition, the relevance of alterations in lipid metabolism in cancer dissemination has been recently highlighted (40).

Herein, we have identified and validated *in vitro* and *in vivo* lipid metabolism features implicated in the differential dissemination of CRC cells to specific niches—mainly to the liver for KM12SM cells and to the lung for KM12L4a cells. Importantly, some of the metabolic differences are worst prognostic factors of CRC patients.

In addition to the Warburg effect and increased glutaminolysis, the role of lipid metabolism in cancer has been recognized. Fatty acids are key structural components of cell membranes. They are substrates for ATP production and key mediators of oncogenic signalling pathways. Our results indicate that changes in lipid metabolism are associated with functional differences between the two metastatic CRC cells and that these changes demand further attention. KM12SM preferentially promotes a higher bioenergetic activity (**Figures 5** **–** **7**) as demonstrated by the increased levels of aerobic glycolysis and oxidative phosphorylation in mitochondria (**Figure 5**). In contrast, KM12L4a is characterized by reduced oxidative phosphorylation and glycolytic performance compared to parental KM12 cells (**Figures 5**, **6**). Nevertheless, when KM12L4a cells are exposed to exogenous FAs, they can increase oxidative phosphorylation to levels similar to those of parental KM12 cells.

In a previous work (40), we compared the cell bioenergetic performance of the isogenic pair non-metastatic SW480 CRC cells and the metastatic SW620 CRC cells with lymph node tropism. SW620 cells showed reduced mitochondrial oxidative phosphorylation without glycolytic changes compared to SW480 cells, indicating an overall energetic advantage. Metastatic SW620 cells had higher levels of reduced glutathione (GSH) compared to isogenic SW480 cells. Interestingly, the increased dependence of KM12L4a cells on extracellular FA uptake could explain the lower oxidative stress observed in this metastatic cell line, as fatty acid oxidation (FAO) can provide NADH and FADH_2_ for reduced glutathione regeneration. In this sense, KM12L4a cells were almost unaffected by metformin, which inhibits respiratory chain complex I, suggesting that this cell line is highly dependent on respiratory chain complex II to obtain energy through lipid metabolism using FADH_2_ obtained during β-oxidation.

Moreover, specialized transporters facilitate the uptake of exogenous FA across the plasma membrane. Whereas normal cells rely on exogenous FA uptake, cancer cells promote *de novo* FA synthesis, independently of the extracellular circulating lipid levels, highlighting the relevance of the crucial role played by FA synthesis in tumorigenesis (41). Here, we have observed, in addition to a dysregulation in the EMT and stemness markers, that metastatic CRC cells differentially reactivated FA uptake to get different metastatic niches in comparison to non-metastatic CRC cells, suggesting that metabolic reprogramming helps dictate metastatic organ colonization. The best-characterized FA receptors/transporters include CD36, also known as fatty acid translocase (FAT), fatty acid transport protein family (FATPs), also known as solute carrier protein family 27 (SLC27), and plasma membrane fatty acid-binding proteins (FABPpm), along with low-density lipoprotein receptor LDLR. Importantly, most of them showed increased gene and protein expression in tumours. These results are also in agreement with the fact that obesity and metabolic disorders increase the risk and worse prognosis of certain types of cancer, including CRC (42).

One of the goals of the study was to determine whether the different FA uptake detected also plays a relevant role in the prognosis of CRC patients. First, through meta-analysis we observed a correlation with worst prognosis of *CD36*, *LDLR*, *FABP4*, *AGPAT1*, and *SCD* overexpression and *FASN* reduction in CRC. High CD36 expression has been correlated with poor prognosis in several tumour types, including breast, ovarian, gastric, and prostate (43, 44). Regarding LDLR, an elevated tumour expression of this protein has been shown to accelerate LDL cholesterol-mediated breast cancer growth in mouse models of hyperlipidaemia (45). Furthermore, LDLR expression and its transcriptional regulation in tumours have been largely unexplored (46). Remarkably, no other studies linking these metabolic abnormalities to CRC prognosis have been previously reported. Furthermore, experiments with plasma from obese volunteers reinforced this idea, further supporting the link between metabolic alterations and CRC prognosis. Remarkably, plasma from obese volunteers produced a significantly increased basal, but not spare, respiratory capacity of metastatic cells, as well as increased tumorigenic and metastatic properties of metastatic cells in contrast to KM12C cells.

Second, through meta-analysis of primary CRC and metastatic *foci*, we also confirmed that *LPL*, *CD36*, *LDLR*, *FASN*, *FABP1*, *SCD*, *ABCA1*, *APOA1*, *AGPAT1*, and *SREBF1* expression levels were associated with lung or liver tropisms, suggesting that metabolic alterations help dictate, among other alterations, specific organotropisms in CRC patients. In this sense, the depletion of CD36 in KM12SM and KM12L4a cells helped to confirm its association to lung metastasis *in vitro*, where a decrease in tumorigenic and metastatic properties was observed, and *in vivo* with a partial impairment of lung metastasis homing of KM12L4a cells in contrast to KM12SM cells, where no alterations in homing were observed. In addition, the comparison of the expression levels of these genes between lung and liver metastatic foci indicated that *LPL*, *CD36*, *LDLR*, *FASN*, *FABP4*, *SCD*, *ABCA1*, and *AGPAT1* were associated with metastatic lung foci; whereas the expression of *FABP1*, *APOA1*, and *SREBF1* was associated with metastatic liver foci (**Figure S4**).

## Conclusion

Here, we have identified metabolic and functional differences in metastatic CRC cells with different tropisms, reflecting the relevance of distinct metabolic adaptations of metastatic CRC cells that may help dictate the organ of colonization. Importantly, the identified dysregulated metabolic proteins have also been shown to be prognostic factors of CRC and potential metastatic markers that show differential correlation with the organs of colonization.

## Data Availability Statement

The datasets presented in this study can be found in online repositories. The names of the repository/repositories and accession number(s) can be found in the article/**Supplementary Material**.

## Ethics Statement

The studies involving human participants were reviewed and approved by the Platform for Clinical Trials in Nutrition and Health (GENYAL) at IMDEA Food Institute (Madrid, Spain). Volunteers included in the GENYAL database were contacted to participate in this study (IMD PI:030). The patients/participants provided their written informed consent to participate in this study. The Ethical Committee of the Instituto de Salud Carlos III (Spain) approved the protocols used for experimental work with mouse after approval for the ethical committee OEBA (Proex 285/19).

## Author Contributions

Conception and design: MG, AM-C, AR, and RB. Development of methodology: AM-C, AQ-F, AP-G, GS-F, and MF-A, IE-S. Performing of research: AM-C, AQ-F, AP-G, GS-F, MF-A, VL-A IE-S and MG. Analysis and interpretation of data: AM-C, AQ-F, AP-G, GS-F, MF-A, VL-A, and MG, AR, and RB. Writing and review of the manuscript: AM-C, MG, AR, and RB. Revision of the manuscript: all authors. Technical, obtaining and processing of samples, or material support: MG, AR, RB, VL-A, and MF-A. All authors contributed to the article and approved the submitted version.

## Funding

This work was supported by grants cofounded by Fondo Europeo de Desarrollo Regional -FEDER- PI17CIII/00045 and PI20CIII/00019 from the AES-ISCIII program to RB from the Instituto de Salud Carlos III (ISCIII) and grants from Spanish Ministry of Science (Plan Nacional I+D+i PID2019-110183RB-C21), Regional Government of Community of Madrid (P2018/BAA-4343-ALIBIRD2020-CM, and Y2020/BIO-6350), and Ramón Areces Foundation (CIVP19A5937) to AR. AM-C FPU predoctoral contract is supported by the Spanish Ministerio de Educación, Cultura y Deporte. AQ-F acknowledges Comunidad de Madrid for the Garantía Juvenil PEJD-2017-PRE/BMD-3394 contract. GS-F is a recipient of a predoctoral contract (grant number 1193818N) supported by the Flanders Research Foundation (FWO).

## Conflict of Interest

The authors declare that the research was conducted in the absence of any commercial or financial relationships that could be construed as a potential conflict of interest.

The handling editor JI-G declared a shared parent affiliation with the author MF-A at the time of review.

## Publisher’s Note

All claims expressed in this article are solely those of the authors and do not necessarily represent those of their affiliated organizations, or those of the publisher, the editors and the reviewers. Any product that may be evaluated in this article, or claim that may be made by its manufacturer, is not guaranteed or endorsed by the publisher.

## Abbreviations

ABCA1, phospholipid-transporting ATPase ABCA1; AGPAT, 1-acyl-sn-glycerol-3-phosphate acyltransferase beta; APOA1, apolipoprotein A-I; BIP (HSPA5), endoplasmic reticulum chaperone BiP; CD29, integrin beta-1; CD36, platelet glycoprotein 4; CD44, CD44 antigen; CD133, prominin-1; CHOP (DDIT3), DNA damage-inducible transcript 3 protein; COX2 (PTGS2), prostaglandin G/H synthase 2; FABP1, fatty acid-binding protein, liver; FABP4, fatty acid-binding protein, adipocyte; FASN, fatty acid synthase; SCD, stearoyl-CoA desaturase 5; ILR6, interleukin-6 receptor subunit alpha; LDLR, low-density lipoprotein receptor; Lgr5, leucine-rich repeat-containing G-protein coupled receptor 5; LPL, lipoprotein lipase; MSI1, RNA-binding protein Musashi homolog 1; SREBF1, sterol regulatory element-binding protein 1; TNFα, tumour necrosis factor.

## References

[1] Chaffer CL Weinberg RA. A Perspective on Cancer Cell Metastasis. Science (2011) 331:1559–64. doi: 10.1126/science.1203543 21436443

[2] Obenauf AC Massague J. Surviving at a Distance: Organ-Specific Metastasis. Trends Cancer (2015) 1:76–91. doi: 10.1016/j.trecan.2015.07.009 28741564 PMC4673677

[3] Hanahan D Weinberg RA. Hallmarks of Cancer: The Next Generation. Cell (2011) 144:646–74. doi: 10.1016/j.cell.2011.02.013 21376230

[4] Lunt SY Vander Heiden MG. Aerobic Glycolysis: Meeting the Metabolic Requirements of Cell Proliferation. Annu Rev Cell Dev Biol (2011) 27:441–64. doi: 10.1146/annurev-cellbio-092910-154237 21985671

[5] Boroughs LK DeBerardinis RJ. Metabolic Pathways Promoting Cancer Cell Survival and Growth. Nat Cell Biol (2015) 17:351–9. doi: 10.1038/ncb3124 PMC4939711 25774832

[6] Metallo CM Gameiro PA Bell EL Mattaini KR Yang J Hiller K. Reductive Glutamine Metabolism by IDH1 Mediates Lipogenesis Under Hypoxia. Nature (2011) 481:380–4. doi: 10.1038/nature10602 PMC3710581 22101433

[7] Schug ZT Peck B Jones DT Zhang Q Grosskurth S Alam IS. Acetyl-CoA Synthetase 2 Promotes Acetate Utilization and Maintains Cancer Cell Growth Under Metabolic Stress. Cancer Cell (2015) 27:57–71. doi: 10.1016/j.ccell.2014.12.002 25584894 PMC4297291

[8] Elstrom RL Bauer DE Buzzai M Karnauskas R Harris MH Plas DR. Akt Stimulates Aerobic Glycolysis in Cancer Cells. Cancer Res (2004) 64:3892–9. doi: 10.1158/0008-5472.CAN-03-2904 15172999

[9] Yang W Zheng Y Xia Y Ji H Chen X Guo F. ERK1/2-Dependent Phosphorylation and Nuclear Translocation of PKM2 Promotes the Warburg Effect. Nat Cell Biol (2012) 14:1295–304. doi: 10.1038/ncb2629 PMC3511602 23178880

[10] Wise DR DeBerardinis RJ Mancuso A Sayed N Zhang XY Pfeiffer HK. Myc Regulates a Transcriptional Program That Stimulates Mitochondrial Glutaminolysis and Leads to Glutamine Addiction. Proc Natl Acad Sci USA (2008) 105:18782–7. doi: 10.1073/pnas.0810199105 PMC2596212 19033189

[11] Carracedo A Cantley LC Pandolfi PP. Cancer Metabolism: Fatty Acid Oxidation in the Limelight. Nat Rev Cancer (2013) 13:227–32. doi: 10.1038/nrc3483 PMC3766957 23446547

[12] Ou J Miao H Ma Y Guo F Deng J Wei X. Loss of Abhd5 Promotes Colorectal Tumor Development and Progression by Inducing Aerobic Glycolysis and Epithelial-Mesenchymal Transition. Cell Rep (2018) 24:2795–7. doi: 10.1016/j.celrep.2018.08.050 PMC6186214 30184511

[13] Gupta SC Hevia D Patchva S Park B Koh W Aggarwal BB. Upsides and Downsides of Reactive Oxygen Species for Cancer: The Roles of Reactive Oxygen Species in Tumorigenesis, Prevention, and Therapy. Antioxidants Redox Signaling (2012) 16:1295–322. doi: 10.1089/ars.2011.4414 PMC3324815 22117137

[14] Cichon MA Radisky DC. ROS-Induced Epithelial-Mesenchymal Transition in Mammary Epithelial Cells is Mediated by NF-kB-Dependent Activation of Snail. Oncotarget (2014) 5:2827–38. doi: 10.18632/oncotarget.1940 PMC4058048 24811539

[15] Lin CC Cheng TL Tsai WH Tsai HJ Hu KH Chang HC. Loss of the Respiratory Enzyme Citrate Synthase Directly Links the Warburg Effect to Tumor Malignancy. Sci Rep (2012) 2:785. doi: 10.1038/srep00785 23139858 PMC3492867

[16] Gunasinghe NP Wells A Thompson EW Hugo HJ. Mesenchymal-Epithelial Transition (MET) as a Mechanism for Metastatic Colonisation in Breast Cancer. Cancer Metastasis Rev (2012) 31:469–78. doi: 10.1007/s10555-012-9377-5 22729277

[17] Jolly MK Jia D Boareto M Mani SA Pienta KJ Ben-Jacob E. Coupling the Modules of EMT and Stemness: A Tunable 'Stemness Window' Model. Oncotarget (2015) 6:25161–74. doi: 10.18632/oncotarget.4629 PMC4694822 26317796

[18] Kuniyasu H Ohmori H Sasaki T Sasahira T Yoshida K Kitadai Y. Production of Interleukin 15 by Human Colon Cancer Cells is Associated With Induction of Mucosal Hyperplasia, Angiogenesis, and Metastasis. Clin Cancer Res (2003) 9:4802–10. 14581351

[19] Li A Varney ML Singh RK. Constitutive Expression of Growth Regulated Oncogene (Gro) in Human Colon Carcinoma Cells With Different Metastatic Potential and its Role in Regulating Their Metastatic Phenotype. Clin Exp Metastasis (2004) 21:571–9. doi: 10.1007/s10585-004-5458-3 15787094

[20] Calon A Espinet E Palomo-Ponce S Tauriello DV Iglesias M Cespedes MV. Dependency of Colorectal Cancer on a TGF-Beta-Driven Program in Stromal Cells for Metastasis Initiation. Cancer Cell (2012) 22:571–84. doi: 10.1016/j.ccr.2012.08.013 PMC3512565 23153532

[21] Morikawa K Walker SM Nakajima M Pathak S Jessup JM Fidler IJ. Influence of Organ Environment on the Growth, Selection, and Metastasis of Human Colon Carcinoma Cells in Nude Mice. Cancer Res (1988) 48:6863–71. 2846163

[22] Barderas R Mendes M Torres S Bartolome RA Lopez-Lucendo M Villar-Vazquez R. In-Depth Characterization of the Secretome of Colorectal Cancer Metastatic Cells Identifies Key Proteins in Cell Adhesion, Migration, and Invasion. Mol Cell Proteomics (2013) 12:1602–20. doi: 10.1074/mcp.M112.022848 PMC3675817 23443137

[23] Mendes M Pelaez-Garcia A Lopez-Lucendo M Bartolome RA Calvino E Barderas R. Mapping the Spatial Proteome of Metastatic Cells in Colorectal Cancer. Proteomics (2017) 17:1700094. doi: 10.1002/pmic.201700094 28861940

[24] Luque-Garcia JL Martinez-Torrecuadrada JL Epifano C Canamero M Babel I Casal JI. Differential Protein Expression on the Cell Surface of Colorectal Cancer Cells Associated to Tumor Metastasis. Proteomics (2010) 10:940–52. doi: 10.1002/pmic.200900441 20049862

[25] Gomez de Cedron M Acin Perez R Sanchez-Martinez R Molina S Herranz J Feliu J. MicroRNA-661 Modulates Redox and Metabolic Homeostasis in Colon Cancer. Mol Oncol (2017) 11:1768–87. doi: 10.1002/1878-0261.12142 PMC5709620 28981199

[26] Pelaez-Garcia A Barderas R Batlle R Vinas-Castells R Bartolome RA Torres S. A Proteomic Analysis Reveals That Snail Regulates the Expression of the Nuclear Orphan Receptor Nuclear Receptor Subfamily 2 Group F Member 6 (Nr2f6) and Interleukin 17 (IL-17) to Inhibit Adipocyte Differentiation. Mol Cell Proteomics (2015) 14:303–15. doi: 10.1074/mcp.M114.045328 PMC4350027 25505127

[27] Solis-Fernandez G Montero-Calle A Martinez-Useros J Lopez-Janeiro A de Los Rios V Sanz R. Spatial Proteomic Analysis of Isogenic Metastatic Colorectal Cancer Cells Reveals Key Dysregulated Proteins Associated With Lymph Node, Liver, and Lung Metastasis. Cells (2022) 11:447. doi: 10.3390/cells11030447 35159257 PMC8834500

[28] Nguyen DX Bos PD Massague J. Metastasis: From Dissemination to Organ-Specific Colonization. Nat Rev Cancer (2009) 9:274–84. doi: 10.1038/nrc2622 19308067

[29] Costa A Scholer-Dahirel A Mechta-Grigoriou F. The Role of Reactive Oxygen Species and Metabolism on Cancer Cells and Their Microenvironment. Semin Cancer Biol (2014) 25:23–32. doi: 10.1016/j.semcancer.2013.12.007 24406211

[30] Sun SY. N-Acetylcysteine, Reactive Oxygen Species and Beyond. Cancer Biol Ther (2010) 9:109–10. doi: 10.4161/cbt.9.2.10583 PMC2854288 19949311

[31] Halasi M Wang M Chavan TS Gaponenko V Hay N Gartel AL. ROS Inhibitor N-Acetyl-L-Cysteine Antagonizes the Activity of Proteasome Inhibitors. Biochem J (2013) 454:201–8. doi: 10.1042/BJ20130282 PMC4322432 23772801

[32] Vial G Detaille D Guigas B. Role of Mitochondria in the Mechanism(s) of Action of Metformin. Front Endocrinol (Lausanne) (2019) 10. doi: 10.3389/fendo.2019.00294 PMC6514102 31133988

[33] Jackson AL Sun W Kilgore J Guo H Fang Z Yin Y. Phenformin has Anti-Tumorigenic Effects in Human Ovarian Cancer Cells and in an Orthotopic Mouse Model of Serous Ovarian Cancer. Oncotarget (2017) 8:100113–27. doi: 10.18632/oncotarget.22012 PMC5725006 29245964

[34] Bhaskaran K Dos-Santos-Silva I Leon DA Douglas IJ Smeeth L. Association of BMI With Overall and Cause-Specific Mortality: A Population-Based Cohort Study of 3.6 Million Adults in the UK. Lancet Diabetes Endocrinol (2018) 6:944–53. doi: 10.1016/S2213-8587(18)30288-2 PMC6249991 30389323

[35] Ouchi N Parker JL Lugus JJ Walsh K. Adipokines in Inflammation and Metabolic Disease. Nat Rev Immunol (2011) 11:85–97. doi: 10.1038/nri2921 21252989 PMC3518031

[36] Weidinger C Ziegler JF Letizia M Schmidt F Siegmund B. Adipokines and Their Role in Intestinal Inflammation. Front Immunol (2018) 9. doi: 10.3389/fimmu.2018.01974 PMC6194904 30369924

[37] Jeon SM Chandel NS Hay N. AMPK Regulates NADPH Homeostasis to Promote Tumour Cell Survival During Energy Stress. Nature (2012) 485:661–5. doi: 10.1038/nature11066 PMC3607316 22660331

[38] Radisky DC Levy DD Littlepage LE Liu H Nelson CM Fata JE. Rac1b and Reactive Oxygen Species Mediate MMP-3-Induced EMT and Genomic Instability. Nature (2005) 436:123–7. doi: 10.1038/nature03688 PMC2784913 16001073

[39] Coussens LM Werb Z. Inflammation and Cancer. Nature (2002) 420:860–7. doi: 10.1038/nature01322 PMC2803035 12490959

[40] Sanchez-Martinez R Cruz-Gil S Gomez de Cedron M Alvarez-Fernandez M Vargas T Molina S. A Link Between Lipid Metabolism and Epithelial-Mesenchymal Transition Provides a Target for Colon Cancer Therapy. Oncotarget (2015) 6:38719–36. doi: 10.18632/oncotarget.5340 PMC4770732 26451612

[41] Chen M Huang J. The Expanded Role of Fatty Acid Metabolism in Cancer: New Aspects and Targets. Precis Clin Med (2019) 2:183–91. doi: 10.1093/pcmedi/pbz017 PMC6770278 31598388

[42] Garcia-Jimenez C Gutierrez-Salmeron M Chocarro-Calvo A Garcia-Martinez JM Castano A de la Vieja A. From Obesity to Diabetes and Cancer: Epidemiological Links and Role of Therapies. Br J Cancer (2016) 114:716–22. doi: 10.1038/bjc.2016.37 PMC4984860 26908326

[43] Calvo D Gomez-Coronado D Suarez Y Lasuncion MA Vega MA. Human CD36 is a High Affinity Receptor for the Native Lipoproteins HDL, LDL, and VLDL. J Lipid Res (1998) 39:777–88. doi: 10.1016/S0022-2275(20)32566-9 9555943

[44] Ladanyi A Mukherjee A Kenny HA Johnson A Mitra AK Sundaresan S. Adipocyte-Induced CD36 Expression Drives Ovarian Cancer Progression and Metastasis. Oncogene (2018) 37:2285–301. doi: 10.1038/s41388-017-0093-z PMC5920730 29398710

[45] Gallagher EJ Zelenko Z Neel BA Antoniou IM Rajan L Kase N. Elevated Tumor LDLR Expression Accelerates LDL Cholesterol-Mediated Breast Cancer Growth in Mouse Models of Hyperlipidemia. Oncogene (2017) 36:6462–71. doi: 10.1038/onc.2017.247 PMC5690879 28759039

[46] Huang J Li L Lian J Schauer S Vesely PW Kratky D. Tumor-Induced Hyperlipidemia Contributes to Tumor Growth. Cell Rep (2016) 15:336–48. doi: 10.1016/j.celrep.2016.03.020 PMC4984953 27050512

