# Supplementary figures and images for: Metabolic Reprogramming Helps to Define Different Metastatic Tropisms in Colorectal Cancer
Source: Front Oncol. 2022 Jul 25;12:903033. doi: 10.3389/fonc.2022.903033 (PMC9358964; doi:10.3389/fonc.2022.903033)

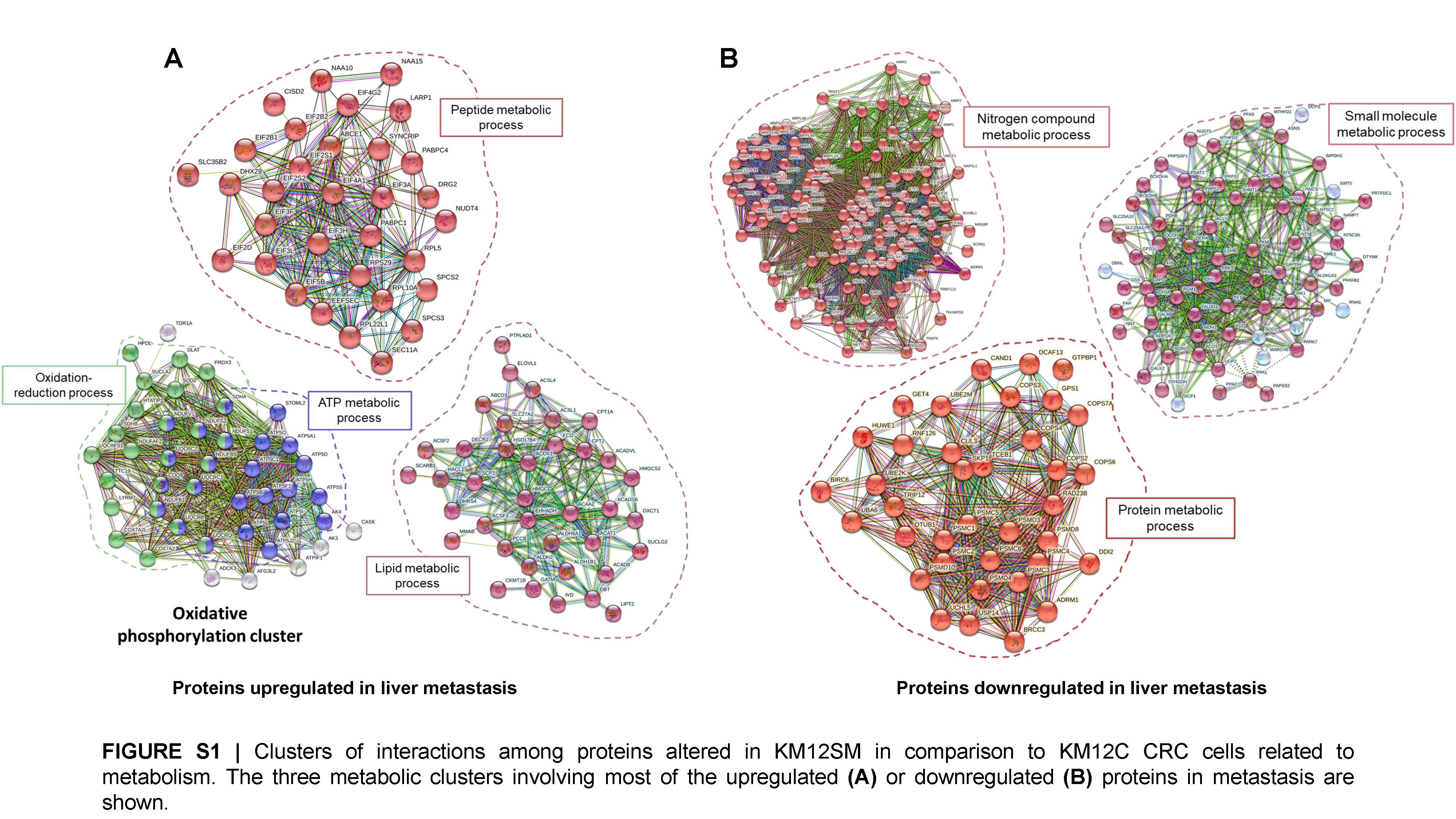

Supplement: Supplementary file 1 [file Image_1.tiff]

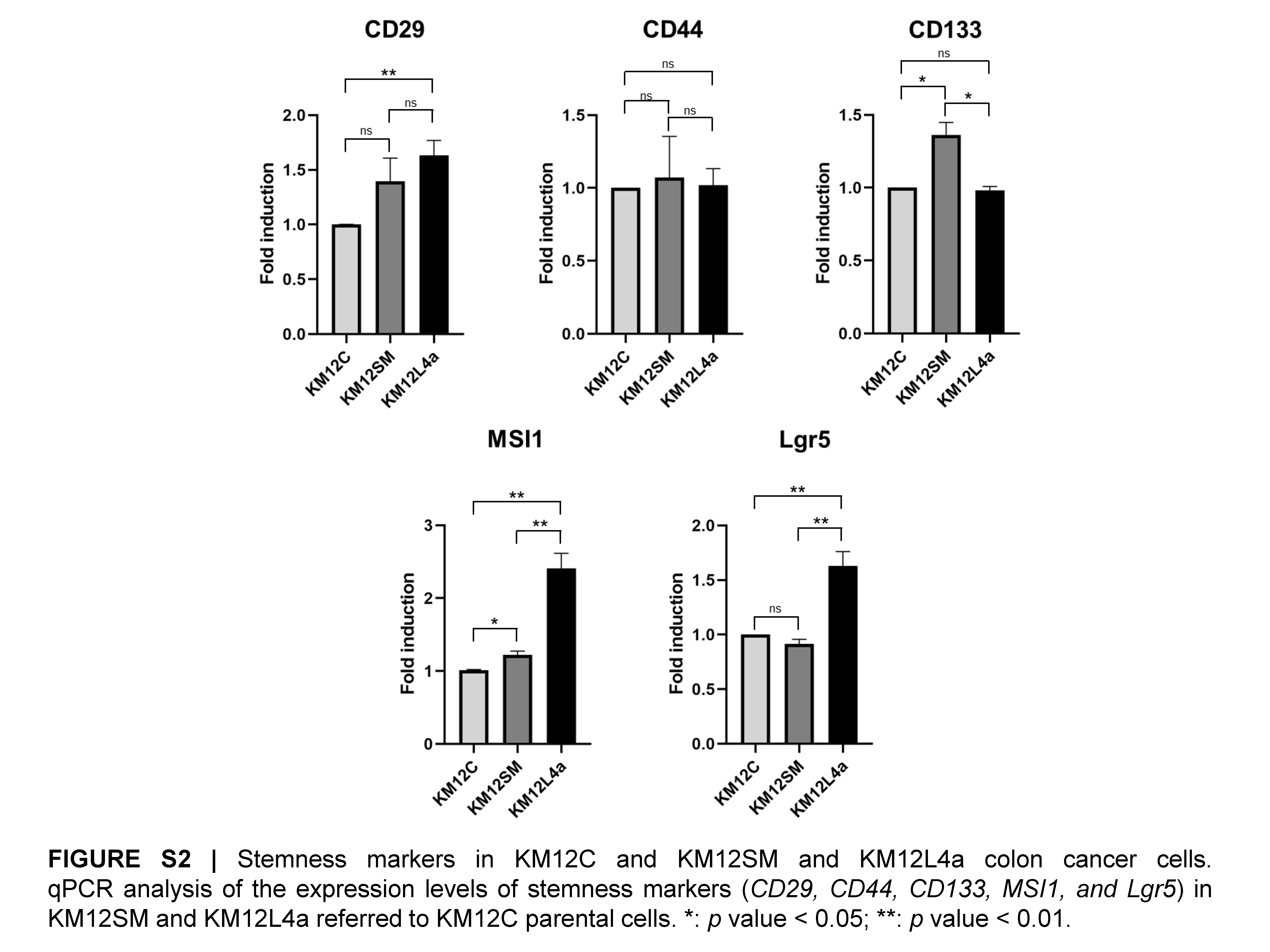

Supplement: Supplementary file 2 [file Image_2.tif]

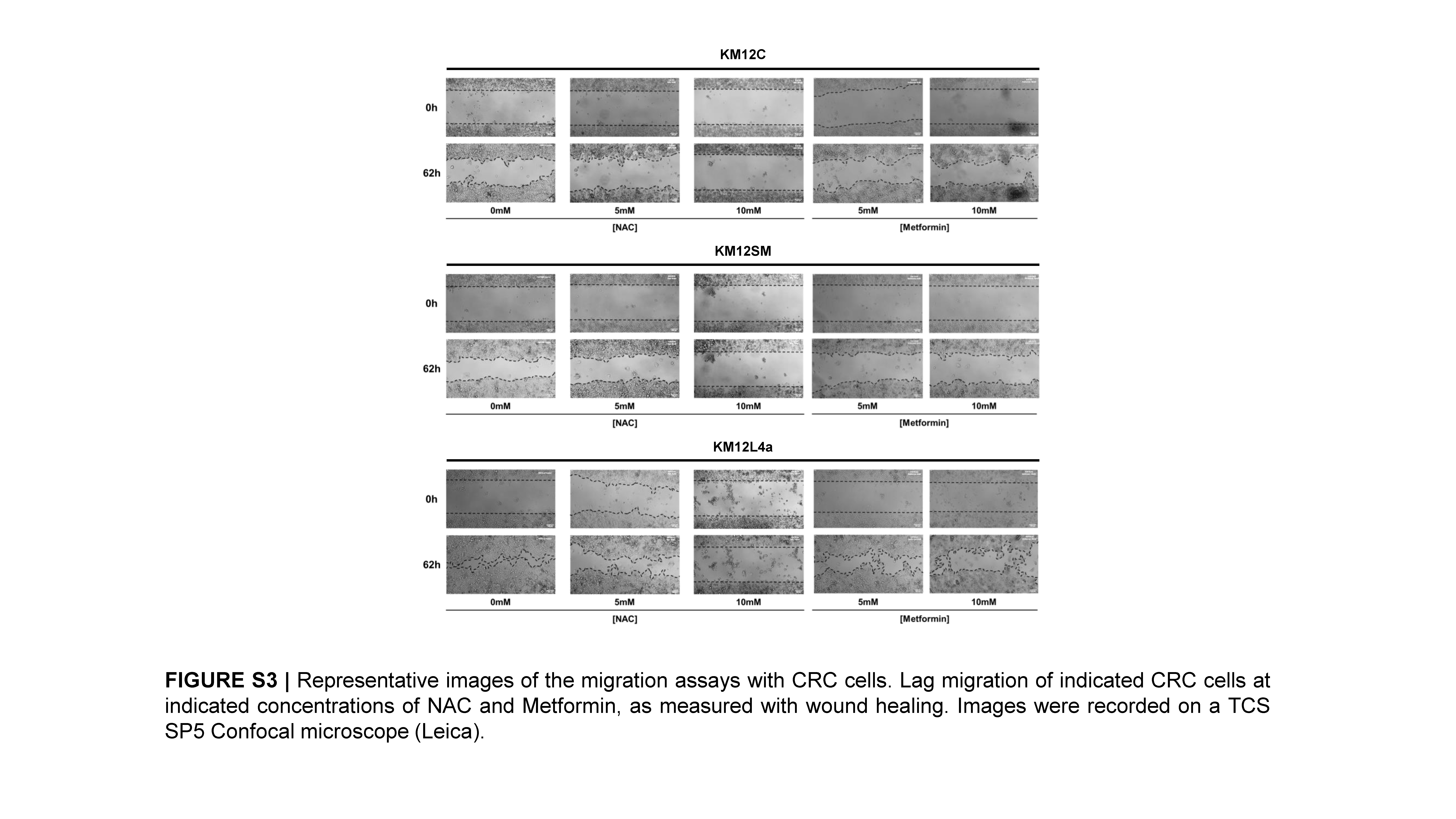

Supplement: Supplementary file 3 [file Image_3.tif]

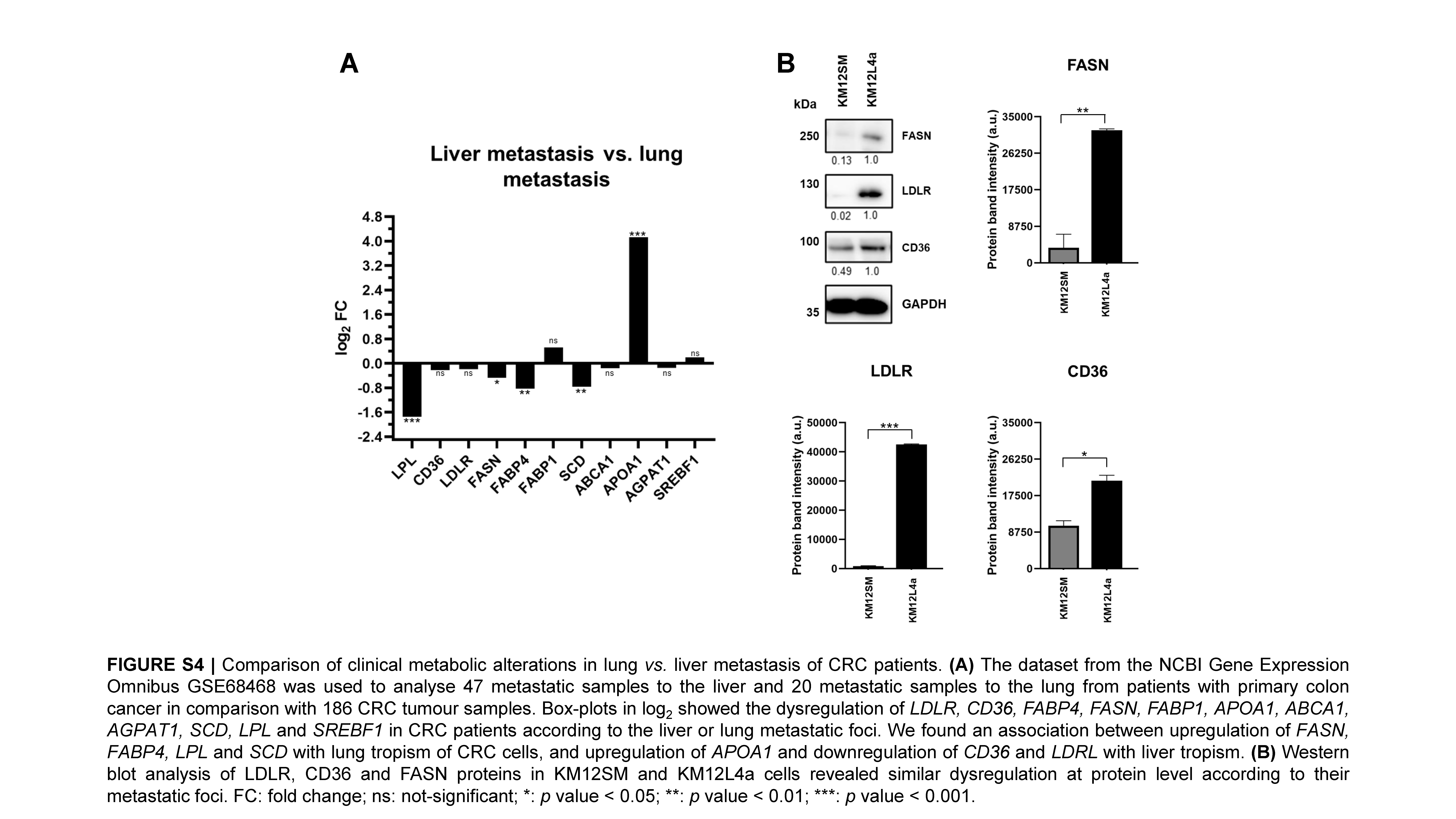

Supplement: Supplementary file 4 [file Image_4.tif]
