# Supplementary material for: Metabolic Reprogramming Helps to Define Different Metastatic Tropisms in Colorectal Cancer
Source: Front Oncol. 2022 Jul 25;12:903033. doi: 10.3389/fonc.2022.903033 (PMC9358964; doi:10.3389/fonc.2022.903033)
Supplement: Supplementary file 7 [file Table_3.docx]

**Table Supplementary 3: Descriptive table of anthropometric data and biochemical data from serum samples**

| **Statistics** | **Sex** | **BMI** | **%Total Fat** | **%Muscle Mass** | **Visceral Fat Classification** | **Waist contour (cm)** | **Systolic blood pressure (mmHg)** | **Diastolic blood pressure (mmHg)** | **Heart rate (bpm)** |
| --- | --- | --- | --- | --- | --- | --- | --- | --- | --- |
| **A** | F:3/M:1 | 21,62(0,21) | 26.35(5.8) | 32.07(5.28) | 4.25(0.5) | 74.42(3.28) | 118.75(15.9) | 74(2.45) | 65.75(5.5) |
| **B** | M:2 | 32,5(1,16) | 43.67(9.7) | 24.97(5.26) | 11(1.63( | 99.32(8.21) | 119.5(6.45) | 76(6.9) | 65.75(9.94) |

| **Statistics** | **Total Cholesterol (mg/dL)** | **Creatinine (mg/dL)** | **Aspartate amino-transferase (UI/L)** | **Alanine amino-transferase (UI/L)** | **HDL cholesterol (mg/dL)** | **LDL cholesterol (mg/dL)** | **Ultrasensitive CRP (mg/dL)** | **Triglycerides (mg/dL)** |
| --- | --- | --- | --- | --- | --- | --- | --- | --- |
| **A** | 204.13(13.11) | 170.40(57.78) | 18.50(8.69) | 18.50(8.69) | 74.23(21.10) | 117.95(20.08) | 0.06(0.051) | 59.75(10.40) |
| **B** | 201.08(21.99) | 106.26(63.8) | 17.75(6.18) | 17.75(6.18) | 52.30(11.02) | 130.18(15.30) | 0.14(0.11) | 93.00(50.18) |
